# *Z*-Selective Synthesis of Trisubstituted Alkenes by the HWE Reaction Using Modified Still–Gennari Type Reagents

**DOI:** 10.1021/acs.joc.5c00470

**Published:** 2025-04-16

**Authors:** Ignacy Janicki

**Affiliations:** Division of Organic Chemistry, Centre of Molecular and Macromolecular Studies, Polish Academy of Sciences, ul. Sienkiewicza 112, 90-363 Łódź, Poland

## Abstract

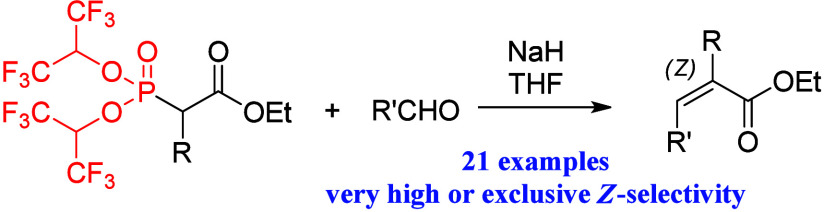

Application of new reagents in the *Z*-selective synthesis of trisubstituted alkenes via modified Horner–Wadsworth–Emmons carbonyl olefination is presented. The reagents tested are ethyl bis(1,1,1,3,3,3-hexafluoroisopropyl)phosphonoalkanoates, structurally similar to Still–Gennari type reagents. A set of various trisubstituted *Z*-alkenes was obtained in very high or quantitative yields with excellent *Z*-selectivity. Remarkably, the procedure was very successful for the synthesis of *Z*-2-aryl-substituted cinnamic acid esters, maintaining exclusive *Z*-selectivity even at an increased temperature.

The carbon–carbon double bond is one of the fundamental structural motifs in organic chemistry. One of the most useful and versatile alkene formation methods is carbonyl olefination using the Horner–Wadsworth–Emmons (HWE) reaction.^1^ Numerous advanced methods for the synthesis of carbon–carbon double bonds were developed, including cross-coupling or metathesis reactions; however, the long-known carbonyl olefination reactions (such as Wittig, HWE, Julia, or Peterson reactions) are still among the most popular.^2^ The key aspect of alkene formation reactions is their stereoselectivity. For thermodynamic reasons, procedures leading to thermodynamically favored *E*-alkenes are already well-developed, while the stereoselective synthesis of *Z*-alkenes can still be problematic. This is why *Z*-selective alkene synthesis is still an important research area. Some of the recent literature reports concerning the synthesis of *Z*-alkenes, including both general methods and more specific reactions for particular applications, are indicated in the references.^3^ These reports include, for example, a practical *Z*-selective, one-pot oxidation and HWE carbonyl olefination reaction sequence reported by Ando et al. that highlights the ongoing interest and practical applicability of the HWE type reactions in organic synthesis.^3h^

In our recent studies, we have presented a new type of reagent for the highly *Z*-selective HWE reaction.^4^ These reagents are structurally similar to the reagents applied in the *Z*-selective Still–Gennari modification of the HWE reaction; however, the phosphorus atom is substituted with two highly electron-withdrawing hexafluoroisopropoxyl groups instead of trifluoroethoxyl groups (Scheme 1).^1c,5a^ The *Z*-selectivity of the HWE reaction is highly correlated with the electron-withdrawing effect of the substituents at the phosphorus atom of the phosphonate reagent; therefore, our modified reagents were expected to exhibit enhanced *Z*-selectivity in the HWE reaction.^6^ This expectation was confirmed in our recent study in which a set of disubstituted *Z*-alkenes was obtained in the course of the reactions of our modified reagents with various aldehydes.^4a^ Moreover, the presented approach has considerable advantages over the popular Still–Gennari olefination procedure, such as the use of a more common, cheaper, and easier to handle base system (simple NaH instead of KHMDS with an 18-crown-6 additive) and milder temperature requirements.

**Scheme 1:**
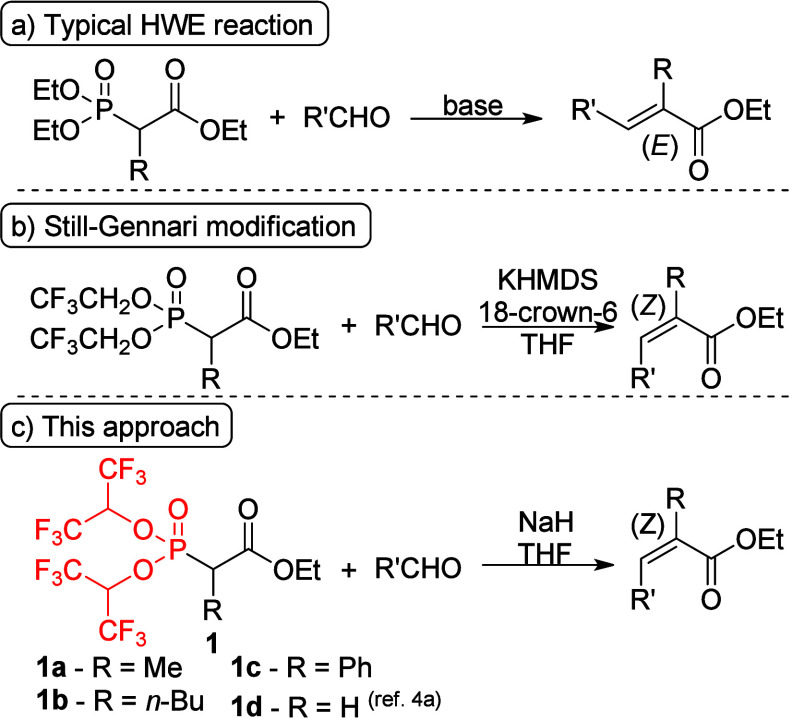
(a) Typical HWE Reaction, (b) *Z*-Selective Still–Gennari Modification of the HWE Reaction, and (c) Our Approach

In this research, application of reagents **1** bearing a bis(1,1,1,3,3,3-hexafluoroisopropyl)phosphonate group for the synthesis of trisubstituted alkenes was investigated (Scheme 1). Stereodefined trisubstituted alkene structural motifs are very important because of their abundance in numerous biologically active compounds and their applications as substrates for further transformations.^2^

The reagents were prepared according to our previously published three-step procedure based on the transformation of diethylphosphonates to bis(1,1,1,3,3,3-hexafluoroisopropyl)phosphonates (Scheme 2).^4b^ This method is an improved version of the previously reported ones, but the main difference is the application of sodium alkoxides instead of appropriate alcohols in the last step.^5b,5c^ Reagents **1a**–**c** were obtained in 63%, 73%, and 51% isolated yields, respectively. The first reagent tested, **1a**, in which R = Me, was chosen for a short optimization study. Several bases were tested (including NaH, *t*-BuOK, NaNH_2_, BuLi, KHMDS, and Cs_2_CO_3_) in the reaction of **1a** with benzaldehyde **2a** to choose the optimal conditions for the reaction. As expected, similarly to our previous observations, NaH was the most effective base. However, in contrast to the reaction of ethyl bis(1,1,1,3,3,3-hexafluoroisopropyl)phosphonoacetate **1d** with benzaldehyde **2a**, in which an excess of NaH significantly decreases the reaction yield, the use of excess NaH was beneficial for the reaction of **1a** with benzaldehyde **2a**. This can be explained by the double deprotonation of **1d** by an excess of a base (leading to a mixture of side products) that cannot take place in the case of **1a**. Therefore, in order to maximize the yields of further reactions, an excess of NaH was used in all of the HWE reactions tested.

**Scheme 2:**
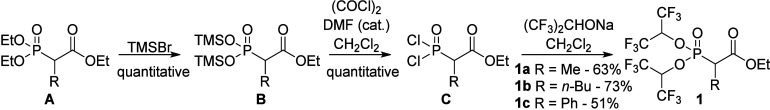
Synthesis of Reagents **1a–c**

Reagent **1a** was tested in HWE olefination reactions with a series of aldehydes **2** (Table 1). Unless otherwise stated, the reactions were typically performed using 2 equiv of NaH in THF from −78 °C to room temperature over 2 h. The reactions did not proceed at −78 °C because under the selected conditions reagents **1** are deprotonated around −40 °C during the slow warming of the reaction mixture. The reactions of **1a** with aromatic aldehydes **2a**–**e** resulted in complete conversion and nearly quantitative isolated yields of unsaturated products **3a**–**e**. Almost complete *Z*-selectivity was observed in all cases. No significant differences were observed between the reactions with variously substituted aromatic aldehydes. Similar results were obtained in the reactions of **1a** with heteroaromatic aldehydes **2f** and **2g** as well as with cyclohexanecarboxaldehyde **2i**. A slightly lower stereoselectivity was observed in the reaction of **1a** with cinnamaldehyde **2h**, but the reaction yield was still very high. Reactions of **1a** with aliphatic aldehydes octanal **2j** and hexanal **2k** resulted in a slightly worse yet still good stereoselectivity (76:24 and 80:20 *Z*:*E* ratios, respectively) and slightly decreased isolated yields (85% and 86%, respectively). Finally, in order to evaluate the utility of the reagents for the olefination of more complex structures, the reaction of **1a** with *N*-Boc-prolinal **2l** was performed. The corresponding reaction using the standard Still–Gennari reagent was reported by Grison et al., who obtained product **3l** in 79% yield as a 90:10 *Z*:*E* mixture (the pure *Z*-product was obtained after column chromatography in 50% isolated yield).^7^ Application of new reagent **1a** resulted in a significantly higher isolated yield of 96% and superior 96:4 *Z*:*E* selectivity. This result proves the high practical potential of the developed reagents.

**Table 1:**
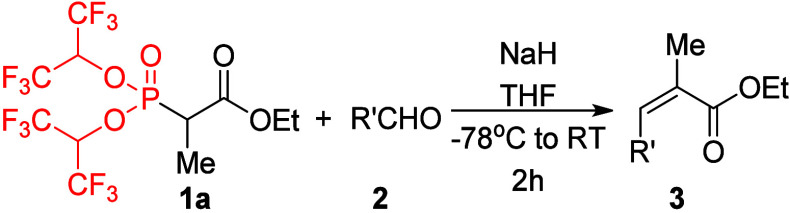
Reactions of **1a** with Aldehydes **2**

entry	aldehyde	product	yield (%)	*Z*:*E*
1	benzaldehyde **2a**	**3a**	99	98:2
2	*p*-tolualdehyde **2b**	**3b**	99	98:2
3	*m*-tolualdehyde **2c**	**3c**	97	98:2
4	*o*-tolualdehyde **2d**	**3d**	98	98:2
5	*p*-nitrobenzaldehyde **2e**	**3e**	97	97:3
6	furfural **2f**	**3f**	95	96:4
7	2-thiophenecarboxaldehyde **2g**	**3g**	99	98:2
8	cinnamaldehyde **2h**	**3h**	99	92:8
9	cyclohexanecarboxaldehyde **2i**	**3i**	98	96:4
10	octanal **2j**	**3j**	85	76:24
11	hexanal **2k**	**3k**	86	80:20
12	*N*-Boc-prolinal **2l**	**3l**	96	96:4

In order to further verify the utility and general applicability of reagents **1** for the synthesis of trisubstituted alkenes with various R groups, reagents bearing *n*-butyl (**1b**) and phenyl (**1c**) R groups were synthesized and tested in the model reactions with selected aldehydes **2**. Despite their simplicity, some of the obtained *Z*-products have not yet been reported in the literature, which highlights the importance of developing new *Z*-selective alkene formation methods. Aldehyde olefination reactions using reagent **1b** proceeded similar to those using **1a**, with a slightly increased stereoselectivity (Table 2). Thus, reactions with benzaldehyde **2a**, *p*-nitrobenzaldehyde **2e**, furfural **2f**, and cinnamaldehyde **2h** resulted in similar yields and *E*:*Z* ratios as the reactions using **1a** with the corresponding aldehydes. Only in the case of the olefination of hexanal **2k** was a slightly better stereoselectivity observed (89:11 compared to 80:20 using **1a**).

**Table 2:**
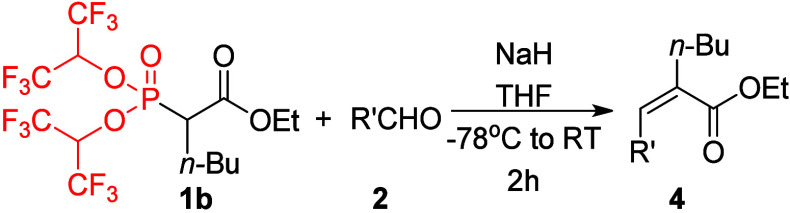
Reactions of **1b** with Aldehydes **2**

entry	aldehyde	product	yield (%)	*Z*:*E*
1	benzaldehyde **2a**	**4a**	98	97:3
2	*p*-nitrobenzaldehyde **2e**	**4b**	96	98:2
3	furfural **2f**	**4c**	96	98:2
4	cinnamaldehyde **2h**	**4d**	99	93:7
5	hexanal **2k**	**4e**	85	89:11

The most interesting results were obtained using reagent **1c** bearing a phenyl R group (Table 3). A comparable study of the standard HWE reaction of ethyl 2-aryl-diethylphosphonoacetates with a series of aromatic aldehydes was performed by Ianni and Waldvogel.^8^ They reported a high-yield, exclusively *E*-selective HWE reaction leading to 2-aryl-substituted cinnamic acid ester derivatives. However, the main drawbacks of these reactions were very long reaction times, 1 week under Masamune–Roush conditions (LiCl and DBU),^9^ and only modest results using standard HWE conditions. By contrast, application of our reagent **1c** in the reaction with a series of aldehydes resulted in the opposite, exclusive *Z*-selectivity and very high yields. The reaction of **1c** with benzaldehyde under the conditions reported above (2 equiv of NaH in THF at −78 °C slowly warmed to room temperature) was much slower than in case of **1a** and **1b**; however, after 24 h, it resulted in 86% of only *Z*-product **5a**. Remarkably, conducting the reaction in refluxing THF overnight resulted in a higher yield (92%) without any loss of stereoselectivity. The reaction of *p*-nitrobenzaldehyde **2e** was much faster. It proceeded smoothly under the standard conditions and resulted in a 96% isolated yield after 2 h. The product of the reaction of **1c** with heteroaromatic aldehyde furfural **2f** was obtained in good 82% yield as sole *Z*-isomer also after 24 h. The reaction of **1c** with cinnamaldehyde **2h** was also exclusively *Z*-selective, and the yield was almost quantitative. However, it required 48 h to reach completion, as after 24 h the reaction yield was 80%.

**Table 3:**
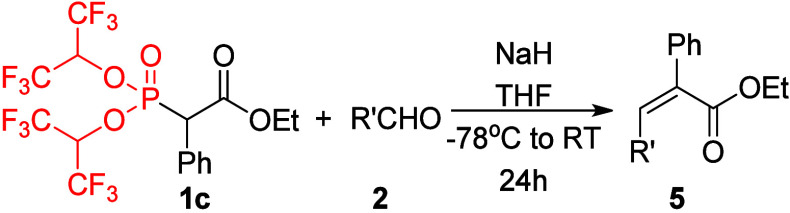
Reactions of **1c** with Aldehydes **2**

entry	aldehyde	product	yield (%)	*Z*:*E*
1	benzaldehyde **2a**	**5a**	86	>99:1
2	benzaldehyde **2a**	**5a**	92^a^	>99:1
3	*p*-nitrobenzaldehyde **2e**	**5b**	96^b^	>99:1
4	furfural **2f**	**5c**	82	>99:1
5	cinnamaldehyde **2h**	**5d**	80	>99:1
6	cinnamaldehyde **2h**	**5d**	98^c^	>99:1
7	hexanal **2k**	**5e**	0	–

a Overnight reaction in refluxing THF.

b Reaction time of 2 h.

c Reaction time of 48 h.

In conclusion, the presented phosphonates **1a**–**c** were proven to be useful, high-yielding, and highly *Z*-selective reagents for the *Z*-selective HWE reaction. The title compounds may be considered as “Still–Gennari type” reagents; however, they exhibit considerable advantages over the standard Still–Gennari reagents, such as the use of a cheaper and easier to handle base (NaH) instead of KHMDS with an 18-crown-6 additive, and the possibility of using them at higher temperatures maintaining the high *Z*-selectivity. Most remarkably, compound **1c** was demonstrated to be perfectly *Z*-selective even when the HWE reaction was conducted overnight in refluxing THF. Moreover, olefination of *N*-Boc-prolinal with **1a** resulted in a considerably better outcome than the same reaction using the standard Still–Gennari approach. In summary, the presented reagents **1** constitute a valuable alternative to Still–Gennari and Ando type reagents, providing a general procedure for obtaining both di- and, particularly importantly, trisubstituted *Z*-alkenes.^1c,5,10^

## Data Availability

The data underlying this study are available in the published article and its Supporting Information.

## Supporting Information Available

General information, procedures, NMR, HRMS, and elemental analysis data, and NMR and HRMS spectra (PDF)
