# Supplementary material for: Z-Selective Synthesis of Trisubstituted Alkenes by the HWE Reaction Using Modified Still–Gennari Type Reagents
Source: J Org Chem. 2025 Apr 16;90(16):5725–8. doi: 10.1021/acs.joc.5c00470 (PMC12038840; doi:10.1021/acs.joc.5c00470)
Supplement: Supplementary file 1 — jo5c00470_si_001.pdf [file jo5c00470_si_001.pdf]

Supporting Information

Z-selective synthesis of trisubstituted alkenes by HWE reaction using modified Still-Gennari type reagents

Ignacy Janicki

Division of Organic Chemistry, Centre of Molecular and Macromolecular Studies, Polish Academy of Sciences,

ul. Sienkiewicza 112, 90-363 Łódź, Poland

email: ignacy.janicki@cbmm.lodz.pl

Table of Contents

General information S2

Procedures S2

NMR, HRMS and elemental analysis data S4

NMR and HRMS spectra S13

References S38

General information

The NMR spectra were recorded using a Bruker Avance Neo 400 spectrometer. Elemental analyses were performed on EuroVector 3018. Mass spectra were recorded with a SYNAPT G2-Si mass spectrometer equipped with an electrospray source and quadrupole-Time-of-Flight mass analyzer (Waters Corp., Milford, MA,USA). All solvents were dried and distilled prior to use. All the starting materials were purchased from Merck, Sigma-Aldrich, TCI Chemicals, or Fluorochem. Reagents **1a**, **1b** and **1c** were prepared according to the procedure reported earlier.[1] All the new compounds were identified by NMR methods and mass spectrometry or elemental analysis. Previously reported substances were identified by ^{1}H NMR.

Procedures

General procedure for the synthesis of 1a-1c.

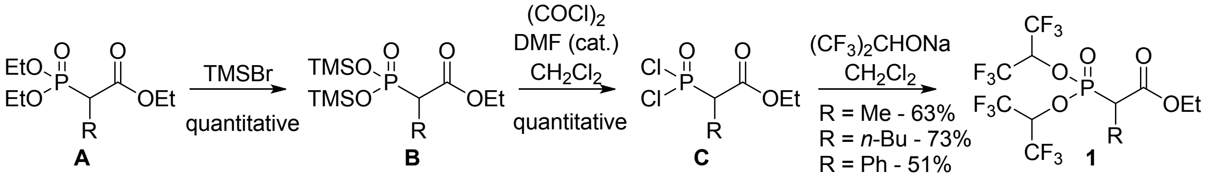

The procedure is similar to the previously developed.[1] In a round-bottom flask under argon atmosphere, TMSBr (30 mmol) was slowly added dropwise at room temperature to diethyl phosphonate **A** (10 mmol). The mixture was stirred for 1 hour, then excess of TMSBr was evaporated under reduced pressure to give bis(trimethylsilyl) phosphonate **B** quantitatively as a colorless liquid. Next it was dissolved in 50 ml of CH_2Cl_2, a catalytic amount of DMF (2–3 drops) was added and oxalyl chloride (30 mmol) was added dropwise to the solution under vigorous stirring accompanied by intense gas evolution. The mixture was stirred for 1 hour at room temperature and then evaporated under reduced pressure to give phosphonic dichloride **C** quantitatively as dark-brown oil. Phosphonic dichloride was dissolved in 50 ml of anhydrous CH_2Cl_2 and solid sodium alkoxide (20 mmol) was added in portions to the solution over 5 minutes. The mixture was stirred for 10 minutes at room temperature, then extracted with distilled water. The water layer was washed with CH_2Cl_2 three times and the combined organic layers were dried using anhydrous $MgSO_4$. Finally, $MgSO_4$ was filtered off, and the solvent was removed under reduced pressure to afford the corresponding phosphonate which was further purified by column chromatography using petroleum ether and ethyl acetate mixture as eluent. Products **1a** and **1b** were isolated as dense, slightly yellow oils in 63% (3.03 g) and 73% (3.82 g) yields and product **1c** was isolated as a slightly yellow solid in 51% (2.80 g) yield.

General procedure for the HWE reaction using reagents 1a-1c.

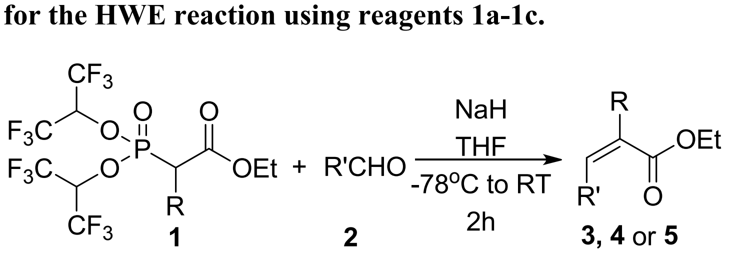

2 mmol of sodium hydride (60% dispersion in mineral oil) was suspended in 3ml of dry THF in a round bottom flask under argon atmosphere. The solution was cooled to −78 °C using acetone-dry ice bath and 1.3 mmol of reagent **1** in 2 mL of dry THF was added. Next 1 mmol of appropriate aldehyde in 5 mL of

THF was added, and the solution was slowly warmed to room temperature. The reaction was quenched typically after 2h in case of reagents **1a** and **1b** or 24h in case of **1c** by addition of 10 ml of saturated NH_4Cl solution. Next the aqueous layer was extracted three times using CH_2Cl_2. The combined organic phases were dried using Na_2SO_4, filtered and evaporated under reduced pressure. The *Z:E* ratios were calculated based on the ^{1}H NMR of the raw products mixture. The products were purified by column chromatography using hexane : ethyl acetate as eluent.

The set of obtained products:

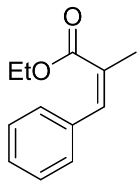

3a 99% 98:2 *Z:E*

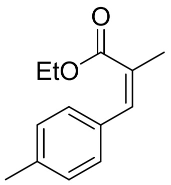

3b 99% 98:2 *Z:E*

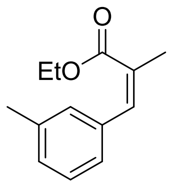

3c 97% 98:2 *Z:E*

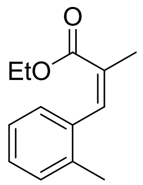

3d 98% 98:2 *Z:E*

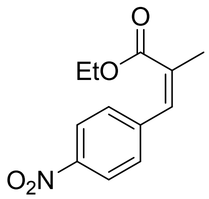

3e 97% 97:3 *Z:E*

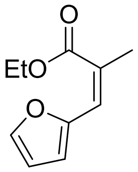

3f 95% 96:4 *Z:E*

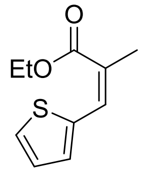

3g 99% 98:2 *Z:E*

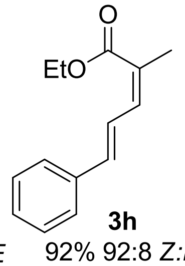

3h 92% 92:8 *Z:E*

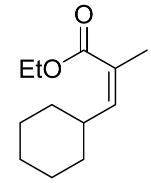

3i 98% 96:4 *Z:E*

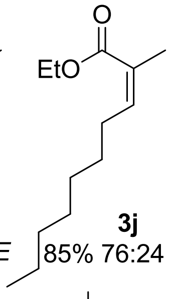

3j 85% 76:24 *Z:E*

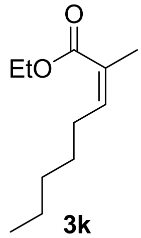

3k 86% 80:20 *Z:E*

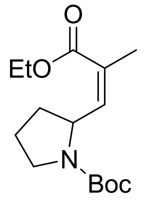

3l 96% 96:4 *Z:E*

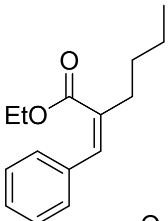

4a 99% 97:3 *Z:E*

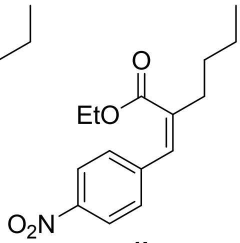

4b 96% 98:2 *Z:E*

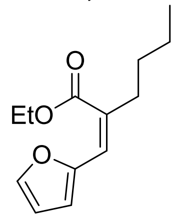

4c 96% 98:2 *Z:E*

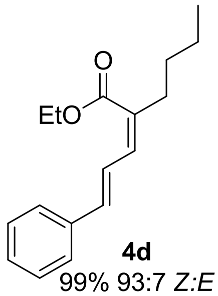

4d 99% 93:7 *Z:E*

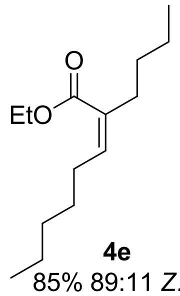

4e 85% 89:11 *Z:E*

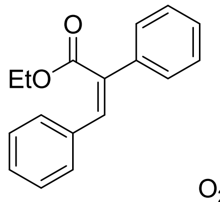

5a 92% >99:1 *Z:E*

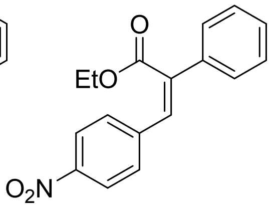

5b 96% >99:1 *Z:E*

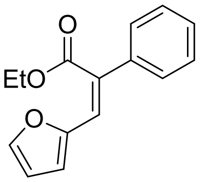

5c 82% >99:1 *Z:E*

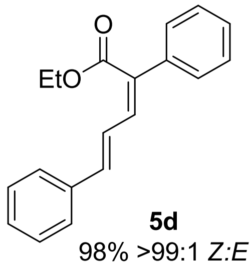

5d 98% >99:1 *Z:E*

NMR, HRMS and elemental analysis data

1a - Ethyl 2-(bis((1,1,1,3,3,3-hexafluoropropan-2-yl)oxy)phosphoryl)propanoate

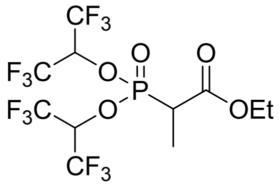

^{1}H NMR (400 MHz, $CDCl_3$) δ 5.30 – 5.11 (m, 2H), 4.32 – 4.15 (m, 2H), 3.27 (dq, *J* = 23.3, 7.4 Hz, 1H), 1.56 (dd, *J* = 20.6, 7.4 Hz, 3H), 1.28 (t, *J* = 7.2 Hz, 3H).

^{13}C{^{1}H} NMR (101 MHz, $CDCl_3$) δ 166.9 (d, *J* = 4.0 Hz), 118.7 (q, *J* = 289.9, 280.2 Hz), 73.2 – 68.1 (m), 62.8, 40.2 (d, *J* = 139.0 Hz), 13.8, 11.2 (d, *J* = 6.6 Hz).

^{31}P{^{1}H} NMR (162 MHz, $CDCl_3$) δ 29.6.

HRMS-ESI (*m/z*): $[M+H]^+$ calculated for $C_{11}H_{12}O_5F_{12}P$, 483.0231, found, 483.0241.

Isolated as slightly yellow oil (3.03 g, 63% yield) after column chromatography on silica gel using ethyl acetate : hexane 1:9 mixture as eluent.

1b - Ethyl 2-(bis((1,1,1,3,3,3-hexafluoropropan-2-yl)oxy)phosphoryl)hexanoate

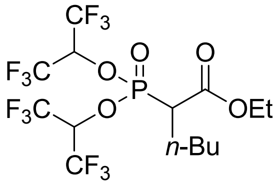

^{1}H NMR (400 MHz, $CDCl_3$) δ 5.25 – 5.07 (m, 2H), 4.32 – 4.16 (m, 2H), 3.14 (ddd, *J* = 22.6, 10.4, 4.0 Hz, 1H), 2.10 – 1.83 (m, 2H), 1.51 – 1.24 (m, 7H), 0.91 (t, *J* = 7.1 Hz, 3H).

^{13}C{^{1}H} NMR (101 MHz, $CDCl_3$) δ 166.3 (d, *J* = 4.5 Hz), 125.1 – 114.7 (m), 71.7 – 69.3 (m), 62.6, 46.0 (d, *J* = 134.3 Hz), 30.1 (d, *J* = 15.2 Hz), 26.1 (d, *J* = 5.5 Hz), 22.1, 13.8, 13.6.

^{31}P{^{1}H} NMR (162 MHz, $CDCl_3$) δ 28.9.

HRMS-ESI (*m/z*): $[M+H]^+$ calculated for $C_{14}H_{18}O_5F_{12}P$, 525.0700, found, 525.0709.

Isolated as slightly yellow oil (3.82 g, 73% yield) after column chromatography on silica gel using ethyl acetate : hexane 1:9 mixture as eluent.

1c - ethyl 2-(bis((1,1,1,3,3,3-hexafluoropropan-2-yl)oxy)phosphoryl)-2-phenylacetate

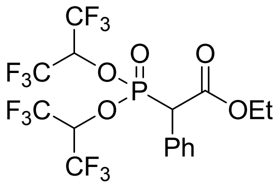

^{1}H NMR (400 MHz, $CDCl_3$) δ 7.46 – 7.35 (m, 5H), 5.37 – 5.00 (m, 2H), 4.49 (d, *J* = 22.8 Hz, 1H), 4.34 – 4.14 (m, 2H), 1.26 (t, *J* = 7.1 Hz, 3H).

^{13}C{^{1}H} NMR (101 MHz, $CDCl_3$) δ 165.8, 129.5 (d, *J* = 7.1 Hz), 129.4 – 128.7 (m), 127.7 (d, *J* = 9.4 Hz), 124.3 – 115.5 (m), 71.9 – 69.0 (m), 63.0, 52.6 (d, *J* = 143.6 Hz), 13.7.

^{31}P{^{1}H} NMR (162 MHz, $CDCl_3$) δ 24.2.

HRMS-ESI (*m/z*): $[M+H]^+$ calculated for $C_{16}H_{14}O_5F_{12}P$, 545.0387, found, 545.0392.

Isolated as slightly yellow amorphous solid (2.80 g, 51% yield) after column chromatography on silica gel using ethyl acetate : hexane 1:19 mixture as eluent.

3a - (*Z*)-ethyl 2-methyl-3-phenylacrylate.

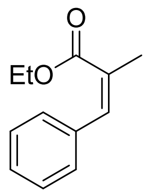

^{1}H NMR (400 MHz, $CDCl_3$) δ 7.35 – 7.22 (m, 5H), 6.73 (s, 1H), 4.13 (q, *J* = 7.2 Hz, 2H), 2.11 (d, *J* = 1.7 Hz, 3H), 1.12 (t, *J* = 7.1 Hz, 3H). [2]

Isolated as colorless oil (188 mg, 98:2 *Z:E* ratio, 99% yield) after column chromatography on silica gel using ethyl acetate : hexane 1:9 mixture as eluent. The ^{1}H NMR spectra is in agreement with the literature data. [2]

3b - (*Z*)-ethyl 2-methyl-3-(*p*-tolyl)acrylate.

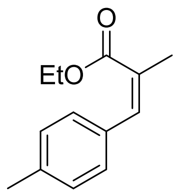

^{1}H NMR (400 MHz, $CDCl_3$) δ 7.21 – 7.06 (m, 4H), 6.66 (s, 1H), 4.14 (q, *J* = 7.1 Hz, 2H), 2.33 (s, 3H), 2.08 (d, *J* = 1.6 Hz, 3H), 1.15 (t, *J* = 7.1 Hz, 3H).

^{13}C{^{1}H} NMR (101 MHz, $CDCl_3$) δ 170.0, 137.6, 134.4, 133.6, 129.4, 128.9, 128.3, 60.7, 21.6, 21.4, 14.0.

HRMS-ESI (*m/z*): $[M+Na]^+$ calculated for $C_{13}H_{16}O_2Na$, 227.1048, found, 227.1046.

Isolated as colorless oil (203 mg, 98:2 *Z:E* ratio, 99% yield) after column chromatography on silica gel using ethyl acetate : hexane 1:9 mixture as eluent.

3c - (*Z*)-ethyl 2-methyl-3-(*m*-tolyl)acrylate.

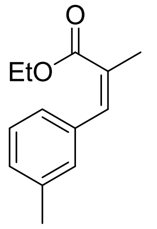

^{1}H NMR (400 MHz, $CDCl_3$) δ 7.22 – 7.14 (m, 1H), 7.09 – 7.01 (m, 3H), 6.67 (s, 1H), 4.12 (q, *J* = 7.1 Hz, 2H), 2.32 (s, 3H), 2.09 (d, *J* = 1.7 Hz, 3H), 1.12 (t, *J* = 7.1 Hz, 3H).

$^{13}C\{^{1}H\}$ NMR (101 MHz, $CDCl_3$) δ 170.0, 137.6, 136.5, 134.3, 130.2, 128.9, 128.4, 128.1, 125.3, 60.7, 21.6, 21.5, 13.9.

Anal. calcd. for $C_{13}H_{16}O_2$: C, 76.4395; H, 7.8952. Found: C, 76.18; H, 7.91.

Isolated as colorless oil (198 mg, 98:2 *Z:E* ratio, 97% yield) after column chromatography on silica gel using ethyl acetate : hexane 1:9 mixture as eluent.

3d - (*Z*)-ethyl 2-methyl-3-(*o*-tolyl)acrylate.

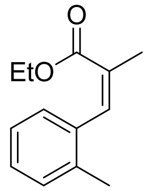

^{1}H NMR (400 MHz, $CDCl_3$) δ 7.22 – 7.04 (m, 4H), 6.83 (s, 1H), 4.00 (q, *J* = 7.1 Hz, 2H), 2.27 (s, 3H), 2.11 (d, *J* = 1.6 Hz, 3H), 0.97 (t, *J* = 7.1 Hz, 3H).

$^{13}C\{^{1}H\}$ NMR (101 MHz, $CDCl_3$) δ 169.2, 136.8, 135.6, 135.5, 130.8, 129.6, 128.3, 127.6, 125.4, 60.4, 21.0, 20.0, 13.8.

HRMS-APCI (*m/z*): $[M+H]^+$ calculated for $C_{13}H_{17}O_2$, 205.1229, found, 205.1234.

Isolated as colorless oil (200 mg, 98:2 *Z:E* ratio, 98% yield) after column chromatography on silica gel using ethyl acetate : hexane 1:9 mixture as eluent.

3e - (*Z*)-ethyl 2-methyl-3-(4-nitrophenyl)acrylate

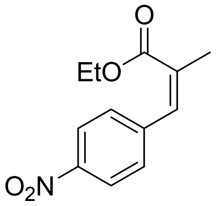

^{1}H NMR (400 MHz, $CDCl_3$) δ 8.20 – 8.12 (m, 2H), 7.41 – 7.35 (m, 2H), 6.74 (s, 1H), 4.12 (q, *J* = 7.1 Hz, 2H), 2.14 (d, *J* = 1.7 Hz, 3H), 1.12 (t, *J* = 7.1 Hz, 3H).

$^{13}C\{^{1}H\}$ NMR (101 MHz, $CDCl_3$) δ 168.6, 147.0, 143.4, 133.9, 132.5, 129.0, 123.5, 61.1, 21.6, 14.0.

HRMS-APCI (*m/z*): $[M+H]^+$ calculated for $C_{12}H_{14}NO_4$, 236.0923, found, 236.0925.

Isolated as slightly yellow amorphous solid (228 mg, 97:3 *Z:E* ratio, 97% yield) after column chromatography on silica gel using ethyl acetate : hexane 1:9 mixture as eluent.

3f - (*Z*)-ethyl 3-(furan-2-yl)-2-methylacrylate

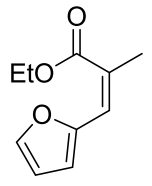

^{1}H NMR (400 MHz, $CDCl_3$) δ 7.37 (d, *J* = 1.7 Hz, 1H), 6.85 (d, *J* = 3.4 Hz, 1H), 6.48 (d, *J* = 1.7 Hz, 1H), 6.40 (dd, *J* = 3.4, 1.8 Hz, 1H), 4.27 (q, *J* = 7.1 Hz, 2H), 2.08 (d, *J* = 1.6 Hz, 3H), 1.32 (t, *J* = 7.2 Hz, 3H).[3]

Isolated as colorless oil (171 mg, 96:4 *Z:E* ratio, 95% yield) after column chromatography on silica gel using ethyl acetate : hexane 1:9 mixture as eluent. The ^{1}H NMR spectra is in agreement with the literature data. [3]

3g - (*Z*)-ethyl 2-methyl-3-(thiophen-2-yl)acrylate

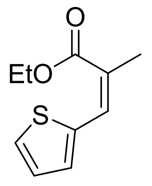

^{1}H NMR (400 MHz, $CDCl_3$) δ 7.35 (d, *J* = 5.1 Hz, 1H), 7.18 (d, *J* = 3.7 Hz, 1H), 6.99 (dd, *J* = 5.2, 3.6 Hz, 1H), 6.90 (s, 1H), 4.29 (q, *J* = 7.1 Hz, 2H), 2.09 (s, 3H), 1.33 (t, *J* = 7.1 Hz, 3H).

$^{13}C\{^{1}H\}$ NMR (101 MHz, $CDCl_3$) δ 168.2, 138.3, 132.2, 130.6, 128.9, 126.4, 124.3, 60.7, 21.7, 14.2.

Anal. calcd. for $C_{10}H_{12}O_2S$: C, 61.1960; H, 6.1627; S, 16.3375. Found: C, 60.98; H, 6.12; S, 16.25.

Isolated as dense milky oil (195 mg, 98:2 *Z:E* ratio, 99% yield) after column chromatography on silica gel using ethyl acetate : hexane 1:9 mixture as eluent.

3h - (2Z,4E)-ethyl 2-methyl-5-phenylpenta-2,4-dienoate

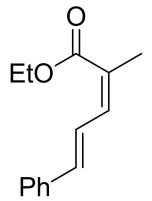

^{1}H NMR (400 MHz, $CDCl_3$) δ 7.92 (dd, *J* = 15.6, 11.3 Hz, 1H), 7.50 – 7.44 (m, 2H), 7.37 – 7.30 (m, 2H), 7.30 – 7.23 (m, 1H), 6.69 (d, *J* = 15.7 Hz, 1H), 6.60 (d, *J* = 11.1 Hz, 1H), 4.27 (q, *J* = 7.1 Hz, 2H), 2.03 (s, 3H), 1.37 (t, *J* = 7.1 Hz, 3H).[4]

Isolated as pale yellow oil (214 mg, 92:8 *Z:E* ratio, 99% yield) after column chromatography on silica gel using ethyl acetate : hexane 1:9 mixture as eluent. The ^{1}H NMR spectra is in agreement with the literature data. [4]

3i - (Z)-ethyl 3-cyclohexyl-2-methylacrylate

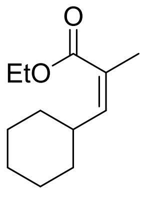

^{1}H NMR (400 MHz, $CDCl_3$) δ 5.71 (d, J = 9.2 Hz, 1H), 4.18 (q, J = 7.1 Hz, 2H), 2.95 – 2.82 (m, 1H), 1.86 (d, J = 1.5 Hz, 3H), 1.76 – 1.60 (m, 5H), 1.38 – 1.23 (m, 5H), 1.22 – 1.12 (m, 1H), 1.09 – 0.96 (m, 2H).[5]

Isolated as colorless oil (192 mg, 96:4 *Z:E* ratio, 98% yield) after column chromatography on silica gel using ethyl acetate : hexane 1:9 mixture as eluent. The ^{1}H NMR spectra is in agreement with the literature data. [5]

3j - (*Z*)-ethyl 2-methyldec-2-enoate

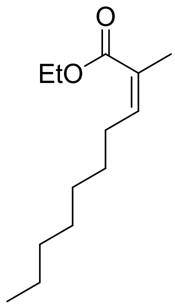

^{1}H NMR (400 MHz, $CDCl_3$) δ 5.91 (t, J = 7.5 Hz, 1H), 4.18 (q, J = 7.1 Hz, 2H), 2.42 (q, J = 7.4 Hz, 2H), 1.88 (s, 3H), 1.49 – 1.19 (m, 13H), 0.87 (t, J = 6.6 Hz, 3H).[6]

Isolated as colorless oil (180 mg, 76:24 *Z:E* ratio, 85% yield) after column chromatography on silica gel using ethyl acetate : hexane 2:98 mixture as eluent. The ^{1}H NMR spectra is in agreement with the literature data. [6] Isolated as 76:24 *Z:E* isomers mixture.

3k - (*Z*)-ethyl 2-methyloct-2-enoate

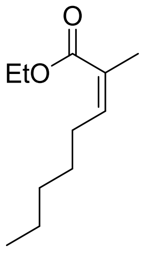

^{1}H NMR (400 MHz, $CDCl_3$) δ 5.96 – 5.88 (m, 1H), 4.19 (q, J = 7.1 Hz, 2H), 2.43 (qd, J = 7.4, 1.4 Hz, 2H), 1.89 (d, J = 1.3 Hz, 3H), 1.45 – 1.35 (m, 2H), 1.30 (t, J = 7.1 Hz, 7H), 0.88 (t, J = 6.9 Hz, 3H).[7]

Isolated as colorless oil (159 mg, 80:20 *Z:E* ratio, 86% yield) after column chromatography on silica gel using ethyl acetate : hexane 2:98 mixture as eluent. The ^{1}H NMR spectra is in agreement with the literature data. [7]

3l - (*S,Z*)-*tert*-butyl 2-(3-ethoxy-2-methyl-3-oxoprop-1-en-1-yl)pyrrolidine-1-carboxylate

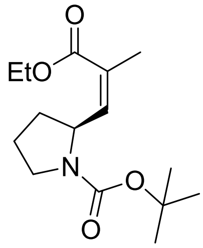

1H NMR (400 MHz, Chloroform-d) δ 5.90 (d, J = 8.1 Hz, 1H), 5.00 (td, J = 7.9, 5.4 Hz, 1H), 4.18 (q, J = 7.1 Hz, 2H), 3.52 – 3.33 (m, 2H), 2.26 (dq, J = 12.8, 7.2 Hz, 1H), 1.89 (s, 3H), 1.87 – 1.77 (m, 2H), 1.72 – 1.63 (m, 1H), 1.39 (s, 9H), 1.29 (t, J = 7.1 Hz, 3H).[8]

Isolated as colorless oil (271 mg, 96:4 *Z:E* ratio, 96% yield) after column chromatography on silica gel using ethyl acetate : hexane 1:4 mixture as eluent. The ^{1}H NMR spectra is in agreement with the literature data. [8]

4a - (*Z*)-ethyl 2-benzylidenehexanoate

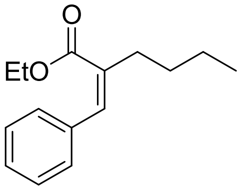

^{1}H NMR (400 MHz, $CDCl_3$) δ 7.33 – 7.19 (m, 5H), 6.62 (s, 1H), 4.13 (q, *J* = 7.1 Hz, 2H), 2.46 – 2.38 (m, 2H), 1.55 – 1.46 (m, 2H), 1.46 – 1.31 (m, 2H), 1.11 (t, *J* = 7.1 Hz, 3H), 0.94 (t, *J* = 7.3 Hz, 3H).

$^{13}C\{^1H\}$ NMR (101 MHz, $CDCl_3$) δ 170.1, 136.4, 135.4, 132.3, 128.07, 128.05, 127.5, 60.6, 35.2, 30.5, 22.2, 13.9, 13.8.

Anal. calcd. for $C_{15}H_{20}O_2$: C, 77.55; H, 8.68. Found: C, 77.43; H, 8.65.

Configuration of the C-C double bond was established based on NOESY experiment.

Isolated as colorless oil (228 mg, 97:3 *Z:E* ratio, 98% yield) after column chromatography on silica gel using ethyl acetate : hexane 1:19 mixture as eluent.

4b (*Z*)-ethyl 2-(4-nitrobenzylidene)hexanoate

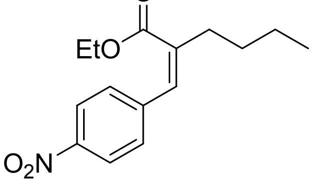

^{1}H NMR (400 MHz, $CDCl_3$) δ 8.19 – 8.11 (m, 2H), 7.41 – 7.34 (m, 2H), 6.64 (s, 1H), 4.13 (q, *J* = 7.1 Hz, 2H), 2.50 – 2.41 (m, 2H), 1.56 – 1.46 (m, 2H), 1.46 – 1.33 (m, 2H), 1.13 (t, *J* = 7.1 Hz, 3H), 0.94 (t, *J* = 7.3 Hz, 3H).

$^{13}C\{^1H\}$ NMR (101 MHz, $CDCl_3$) δ 168.9, 146.9, 143.3, 139.2, 130.2, 128.8, 123.4, 61.0, 35.2, 30.3, 22.2, 13.9.

Anal. calcd. for $C_{15}H_{19}NO_4$: C, 64.97; H, 6.91; N, 5.05. Found: C, 65.02; H, 6.89; N, 5.09.

Configuration of the C-C double bond was established based on NOESY experiment.

Isolated as yellow oil (266 mg, 98:2 *Z:E* ratio, 96% yield) after column chromatography on silica gel using ethyl acetate : hexane 1:19 mixture as eluent. The product slowly isomerize to *E* isomer at room temperature.

4c (*Z*)-ethyl 2-(furan-2-ylmethylene)hexanoate

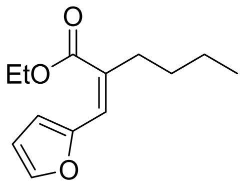

^1H NMR (400 MHz, $CDCl_3$) δ 7.35 (d, *J* = 1.9 Hz, 1H), 6.64 (d, *J* = 3.4 Hz, 1H), 6.38 (dd, *J* = 3.5, 1.8 Hz, 1H), 6.36 (s, 1H), 4.29 (q, *J* = 7.2 Hz, 2H), 2.42 – 2.34 (m, 2H), 1.54 – 1.43 (m, 2H), 1.42 – 1.27 (m, 5H), 0.92 (t, *J* = 7.3 Hz, 3H).

$^{13}C\{^1H\}$ NMR (101 MHz, $CDCl_3$) δ 169.3, 150.8, 142.5, 131.6, 120.5, 111.6, 111.4, 60.7, 35.1, 31.2, 22.2, 14.3, 13.9.

Anal. calcd. for $C_{13}H_{18}O_3$: C, 70.24; H, 8.16; Found: C, 70.24; H, 8.09.

Configuration of the C-C double bond was established based on NOESY experiment.

Isolated as colorless oil (213 mg, 98:2 *Z:E* ratio, 96% yield) after column chromatography on silica gel using ethyl acetate : hexane 1:19 mixture as eluent.

4d (*Z*)-ethyl 2-((*E*)-3-phenylallylidene)hexanoate

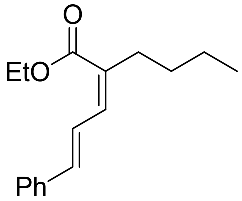

^1H NMR (400 MHz, $CDCl_3$) δ 7.83 (dd, *J* = 15.6, 11.2 Hz, 1H), 7.46 (d, *J* = 7.6 Hz, 2H), 7.33 (t, *J* = 7.1 Hz, 2H), 7.29 – 7.20 (m, 1H), 6.70 (d, *J* = 15.6 Hz, 1H), 6.54 (d, *J* = 11.2 Hz, 1H), 4.28 (q, *J* = 7.1 Hz, 2H), 2.37 (t, *J* = 7.5 Hz, 2H), 1.51 – 1.41 (m, 2H), 1.41 – 1.24 (m, 5H), 0.92 (t, *J* = 7.2 Hz, 3H).

$^{13}C\{^1H\}$ NMR (101 MHz, $CDCl_3$) δ 167.7, 139.2, 137.8, 137.0, 131.8, 128.7, 128.3, 127.1, 126.1, 60.3, 34.5, 31.6, 22.4, 14.4, 14.0.

Anal. calcd. for $C_{17}H_{22}O_2$: C, 79.03; H, 8.58; Found: C, 78.95; H, 8.61.

Configuration of the C-C double bond was established based on NOESY experiment.

Isolated as colorless oil (255 mg, 93:7 *Z:E* ratio, 99% yield) after column chromatography on silica gel using ethyl acetate : hexane 1:19 mixture as eluent.

4e (*Z*)-ethyl 2-butyloct-2-enoate

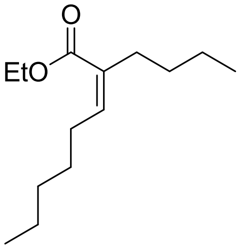

^{1}H NMR (400 MHz, $CDCl_3$) δ 5.82 (t, *J* = 7.5 Hz, 1H), 4.20 (q, *J* = 7.1 Hz, 2H), 2.37 (q, *J* = 7.4 Hz, 2H), 2.23 (t, *J* = 7.4 Hz, 2H), 1.48 – 1.21 (m, 13H), 0.93 – 0.85 (m, 6H).[9]

Isolated as colorless oil (192 mg, 89:11 *Z:E* ratio, 85% yield) after column chromatography on silica gel using ethyl acetate : hexane 1:19 mixture as eluent. The ^{1}H NMR spectra is in agreement with the literature data. [9]

5a (*Z*)-ethyl 2,3-diphenylacrylate

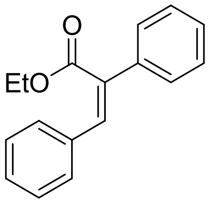

^{1}H NMR (400 MHz, $CDCl_3$) δ 7.54 – 7.46 (m, 2H), 7.43 – 7.27 (m, 8H), 7.05 (s, 1H), 4.28 (q, *J* = 7.1 Hz, 2H), 1.21 (t, *J* = 7.1 Hz, 3H).[10]

Isolated as colorless oil (216 mg, >99:1 *Z:E* ratio, 86% yield after 24h at RT, or 232 mg, >99:1 *Z:E* ratio, 92% yield after overnight reaction in refluxing THF) after column chromatography on silica gel using ethyl acetate : hexane 1:19 mixture as eluent. The ^{1}H NMR spectra is in agreement with the literature data. [10]

5b (*Z*)-ethyl 3-(4-nitrophenyl)-2-phenylacrylate

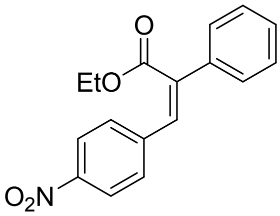

^{1}H NMR (400 MHz, $CDCl_3$) δ 8.26 – 8.18 (m, 2H), 7.59 – 7.51 (m, 2H), 7.51 – 7.46 (m, 2H), 7.45 – 7.35 (m, 3H), 7.05 (s, 1H), 4.29 (q, *J* = 7.1 Hz, 2H), 1.22 (t, *J* = 7.1 Hz, 3H).[10]

Isolated as amorphous white solid (286 mg, >99:1 *Z:E* ratio, 96% yield after 2h) after column chromatography on silica gel using ethyl acetate : hexane 1:19 mixture as eluent. The ^{1}H NMR spectra is in agreement with the literature data. [10]

5c (*Z*)-ethyl 3-(furan-2-yl)-2-phenylacrylate

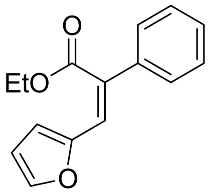

^{1}H NMR (400 MHz, $CDCl_3$) δ 7.47 – 7.41 (m, 2H), 7.41 – 7.28 (m, 3H), 6.77 (s, 1H), 6.55 (d, J = 3.4 Hz, 1H), 6.44 (dd, J = 3.4, 1.8 Hz, 1H), 4.43 (q, J = 7.1 Hz, 2H), 1.37 (t, J = 7.1 Hz, 3H).

^{13}C{^{1}H} NMR (101 MHz, $CDCl_3$) δ 169.2, 150.8, 143.4, 136.1, 131.4, 128.8, 128.2, 125.9, 117.0, 112.3, 111.9, 61.4, 14.2.

Anal. calcd. for $C_{15}H_{14}O_3$: C, 74.36; H, 5.82; Found: C, 74.35; H, 5.84.

Configuration of the C-C double bond was established based on NOESY experiment.

Isolated as amorphous white solid (199 mg, >99:1 *Z:E* ratio, 82% yield) after column chromatography on silica gel using dichloromethane : hexane 3:1 in gradient to 1:1 mixture as eluent.

5d (2*Z*,4*E*)-ethyl 2,5-diphenylpenta-2,4-dienoate

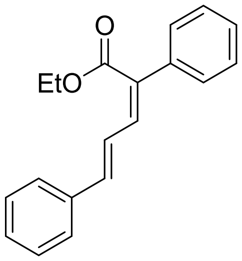

^{1}H NMR (400 MHz, $CDCl_3$) δ 7.62 (dd, J = 15.6, 11.3 Hz, 1H), 7.53 – 7.46 (m, 2H), 7.45 – 7.25 (m, 8H), 6.91 – 6.81 (m, 2H), 4.38 (q, J = 7.1 Hz, 2H), 1.38 (t, J = 7.1 Hz, 3H).[10]

Isolated as colorless oil (222 mg, >99:1 *Z:E* ratio, 80% yield after 24h at RT, or 272 mg, >99:1 *Z:E* ratio, 98% yield after 48h) after column chromatography on silica gel using ethyl acetate : hexane 1:19 mixture as eluent. The ^{1}H NMR spectra is in agreement with the literature data. [10]

NMR and HRMS spectra

1a

^{1}H NMR (400 MHz, $CDCl_3$)

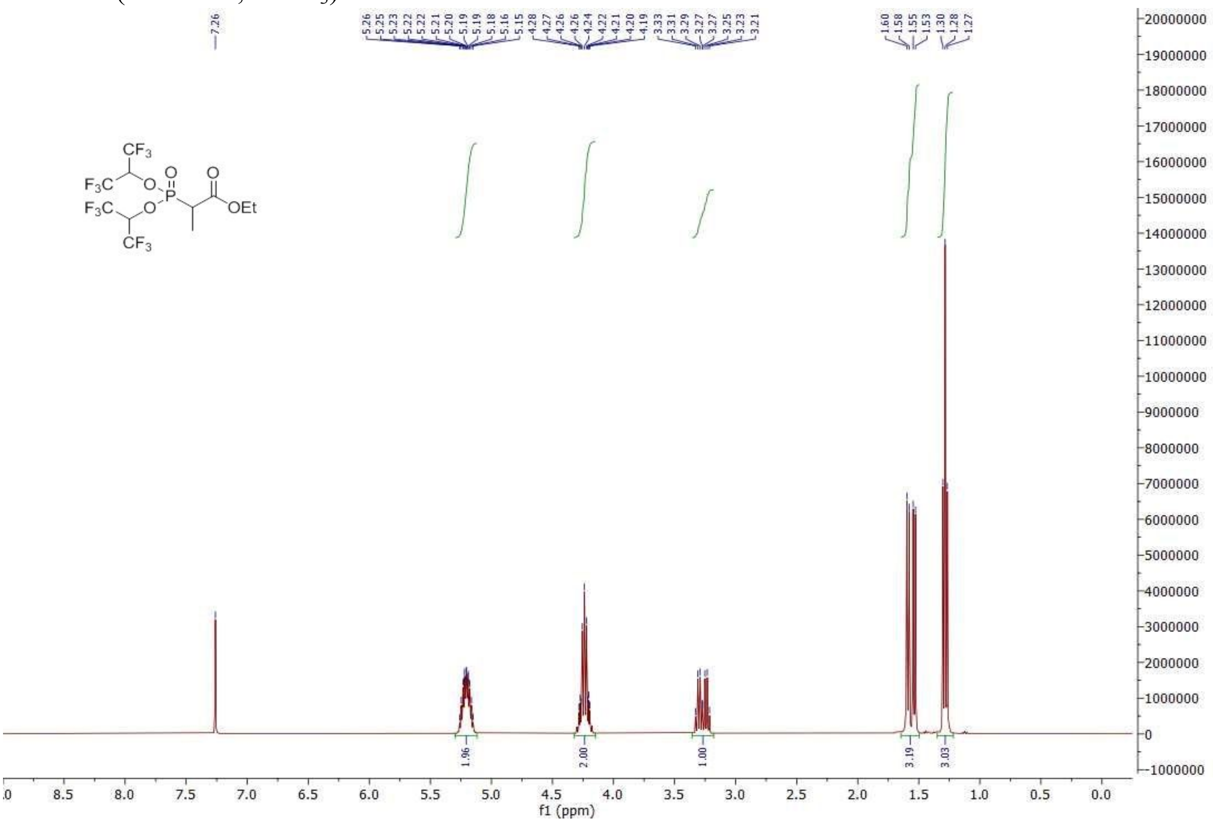

$^{13}C\{^{1}H\}$ NMR (101 MHz, $CDCl_3$)

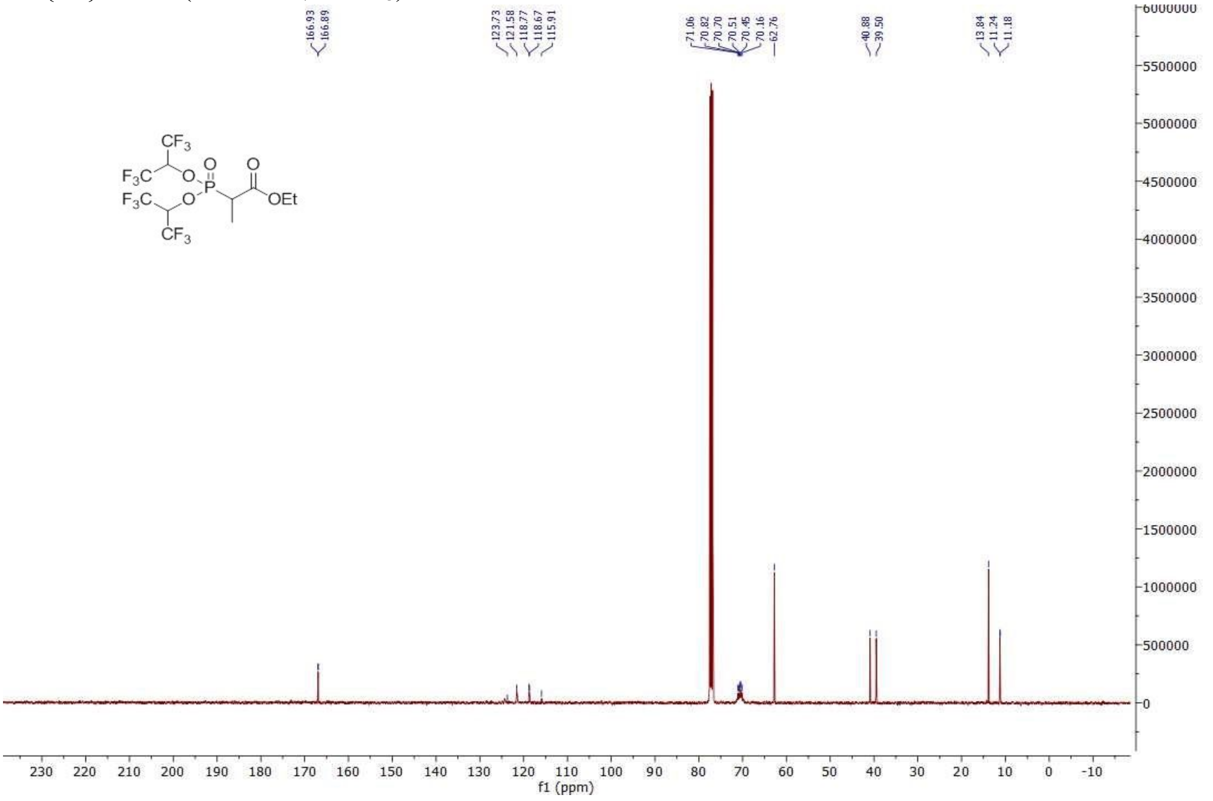

$^{31}P\{^{1}H\}$ NMR (162 MHz, $CDCl_3$)

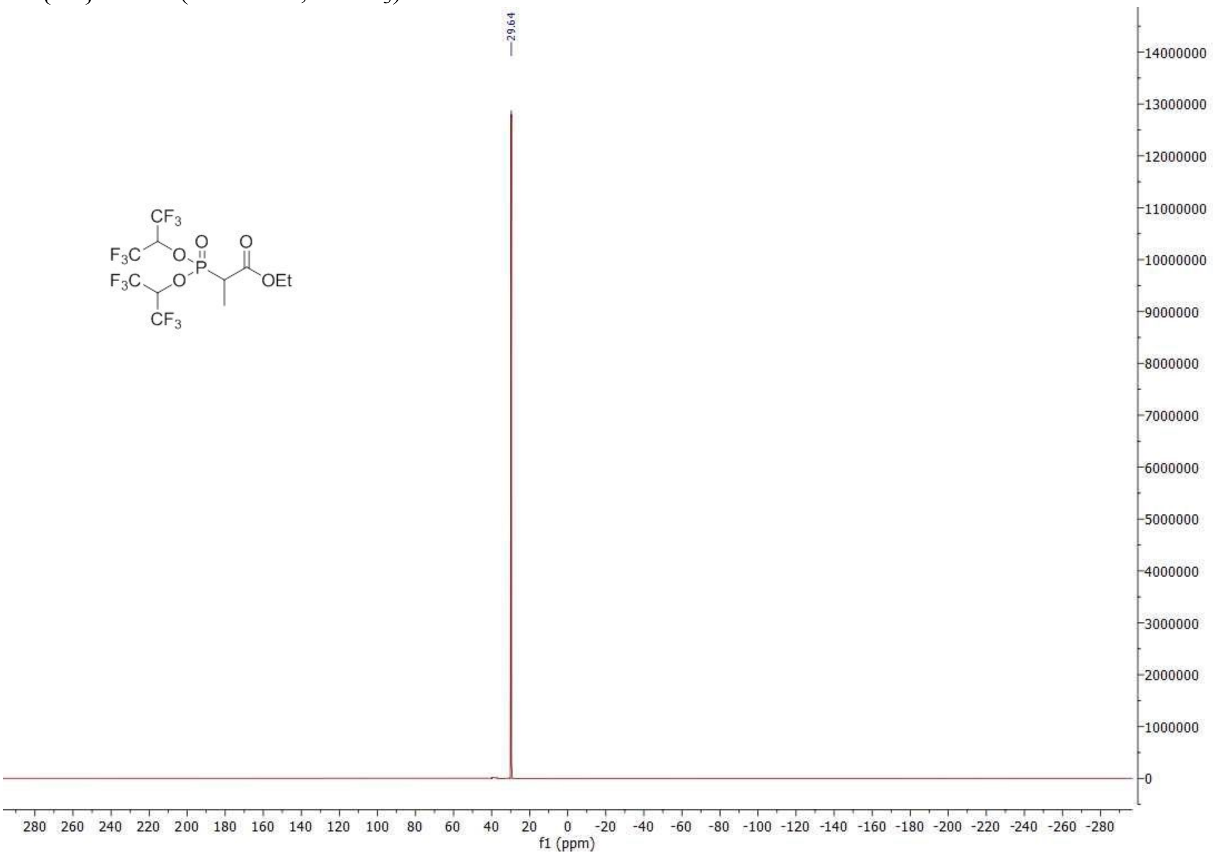

HRMS

Elemental Composition Report **Page 1**

Single Mass Analysis
Tolerance = 5.0 PPM / DBE: min = -1.5, max = 90.0
Element prediction: Off
Number of isotope peaks used for i-FIT = 9

Monoisotopic Mass, Odd and Even Electron Ions
117 formula(e) evaluated with 2 results within limits (all results (up to 1000) for each mass)
Elements Used:
C: 0-15 H: 0-15 O: 0-6 F: 0-15 P: 1-1
230425_SG_CA 19 (0.287) Cm (19:22-1:6)

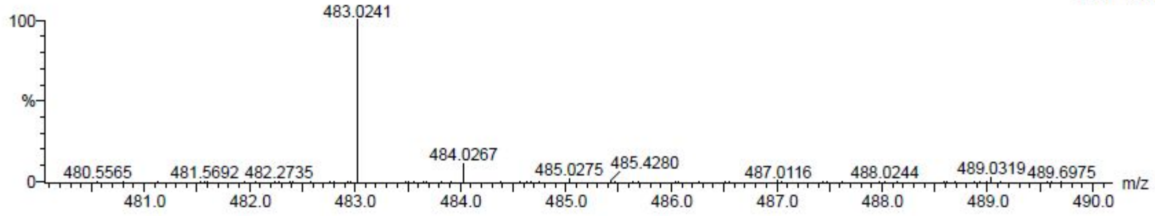

Mass	Calc. Mass	mDa	PPM	DBE	i-FIT	Norm	Conf(%)	Formula
Minimum:				-1.5				
Maximum:		5.0	5.0	90.0				
483.0241	483.0231	1.0	2.1	0.5	361.5	0.102	90.29	C11 H12 O5 F12 P
	483.0219	2.2	4.6	4.5	363.7	2.332	9.71	C14 H11 O4 F11 P

1b

^{1}H NMR (400 MHz, $CDCl_3$)

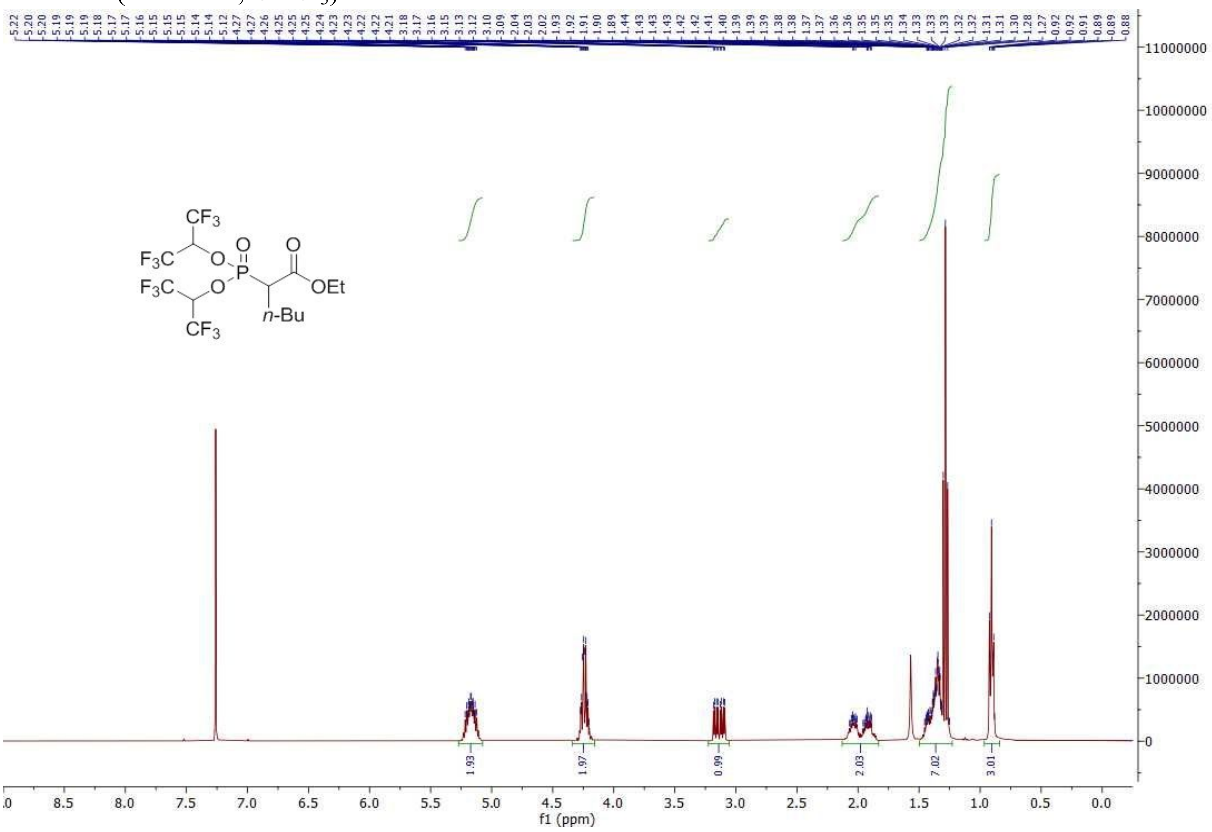

$^{13}C\{^{1}H\}$ NMR (101 MHz, $CDCl_3$)

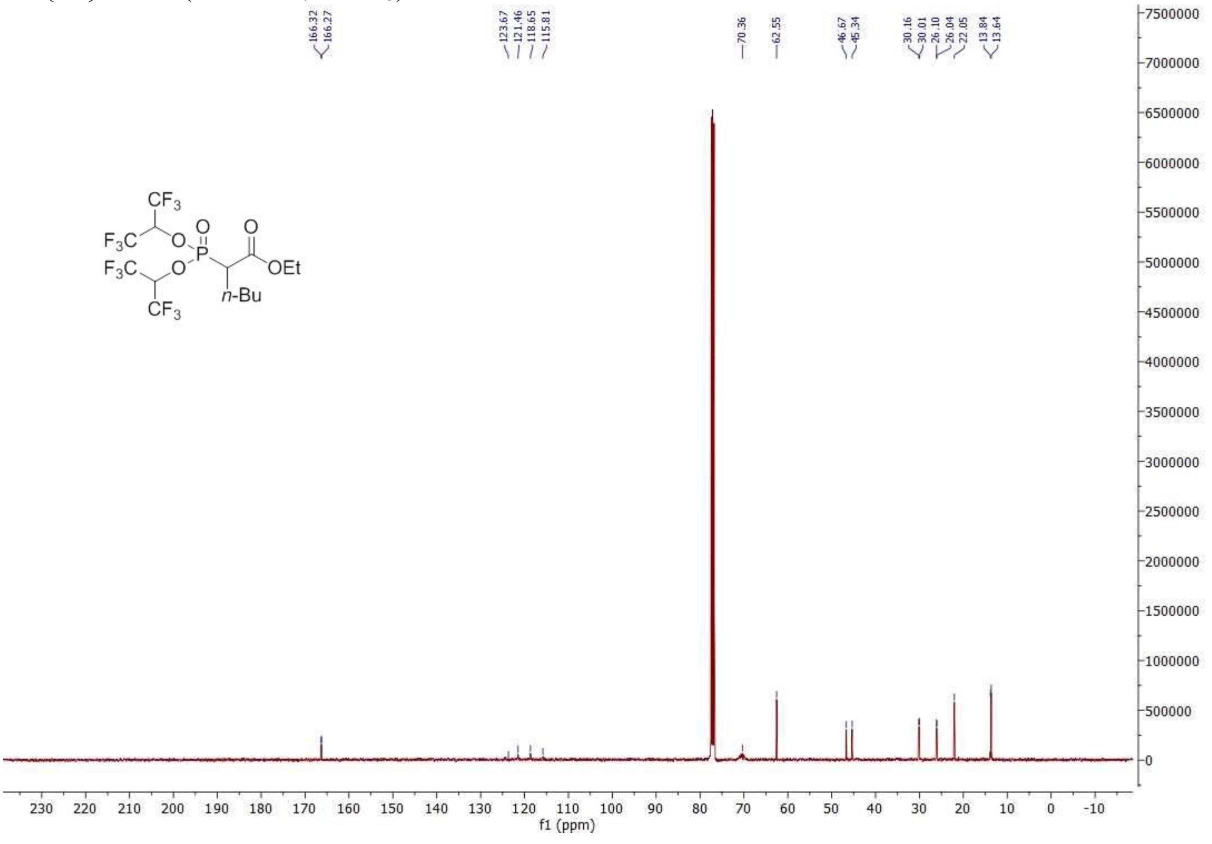

$^{31}P\{^{1}H\}$ NMR (162 MHz, $CDCl_3$)

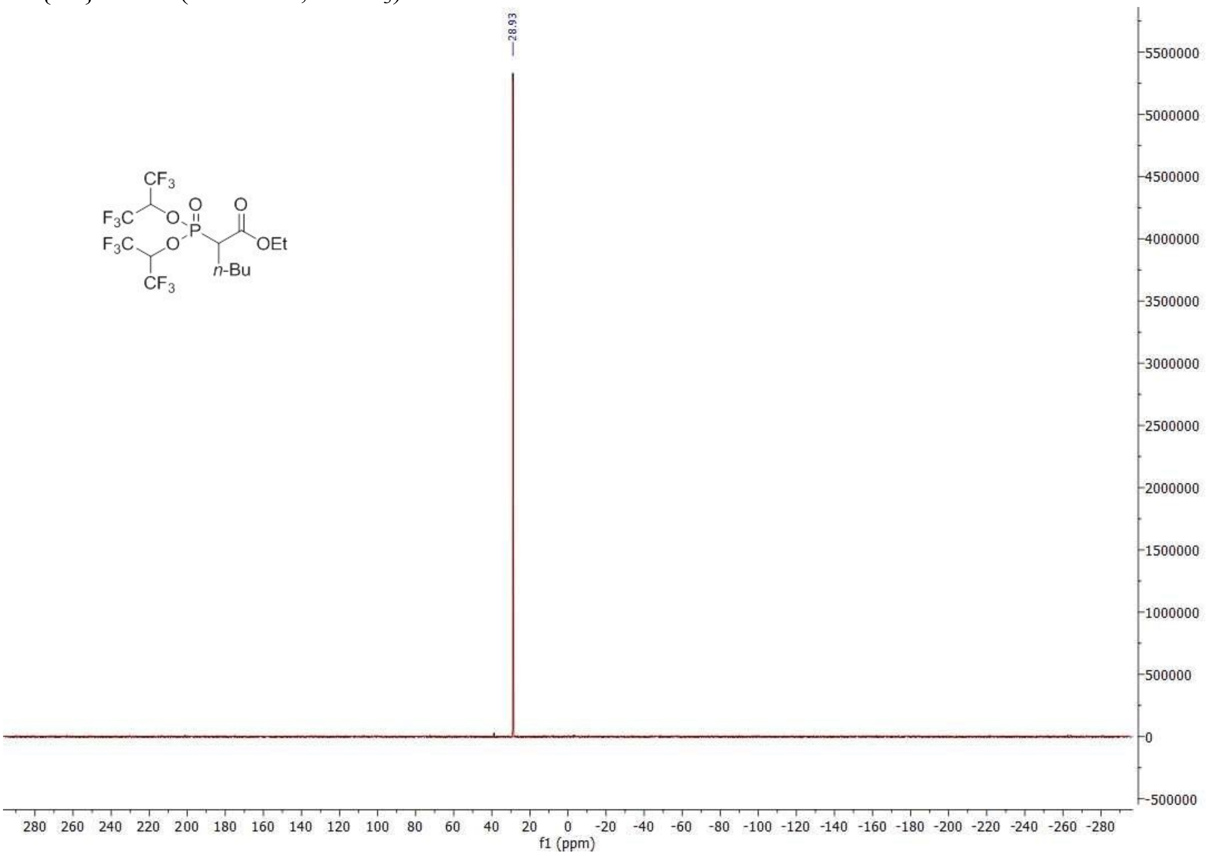

HRMS

Elemental Composition Report **Page 1**

Single Mass Analysis
Tolerance = 5.0 PPM / DBE: min = -1.5, max = 90.0
Element prediction: Off
Number of isotope peaks used for i-FIT = 9

Monoisotopic Mass, Odd and Even Electron Ions
145 formula(e) evaluated with 4 results within limits (all results (up to 1000) for each mass)
Elements Used:
C: 0-20 H: 1-20 O: 0-7 F: 0-15 P: 1-1
230607_SG_DA 23 (0.321) Cm (23:25-1:10)

TOF MS ES+
9.30e+006

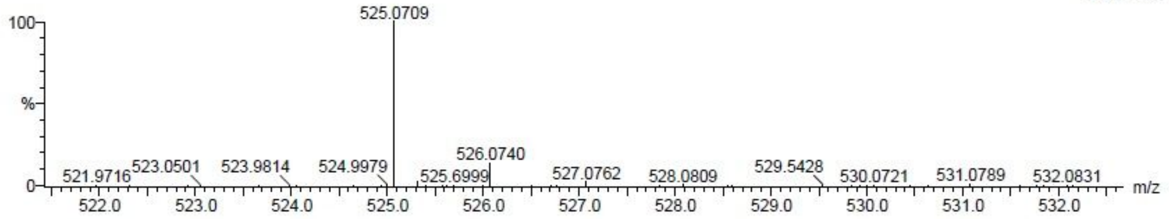

Mass	Calc. Mass	mDa	PPM	DBE	i-FIT	Norm	Conf(%)	Formula
Minimum:				-1.5				
Maximum:		5.0	5.0	90.0				
525.0709	525.0700	0.9	1.7	0.5	354.0	0.253	77.63	C14 H18 O5 F12 P
	525.0689	2.0	3.8	4.5	355.3	1.556	21.09	C17 H17 O4 F11 P
	525.0725	-1.6	-3.0	3.5	358.5	4.706	0.90	C16 H19 O7 F9 P
	525.0713	-0.4	-0.8	7.5	359.4	5.585	0.38	C19 H18 O6 F8 P

1c

^{1}H NMR (400 MHz, $CDCl_3$)

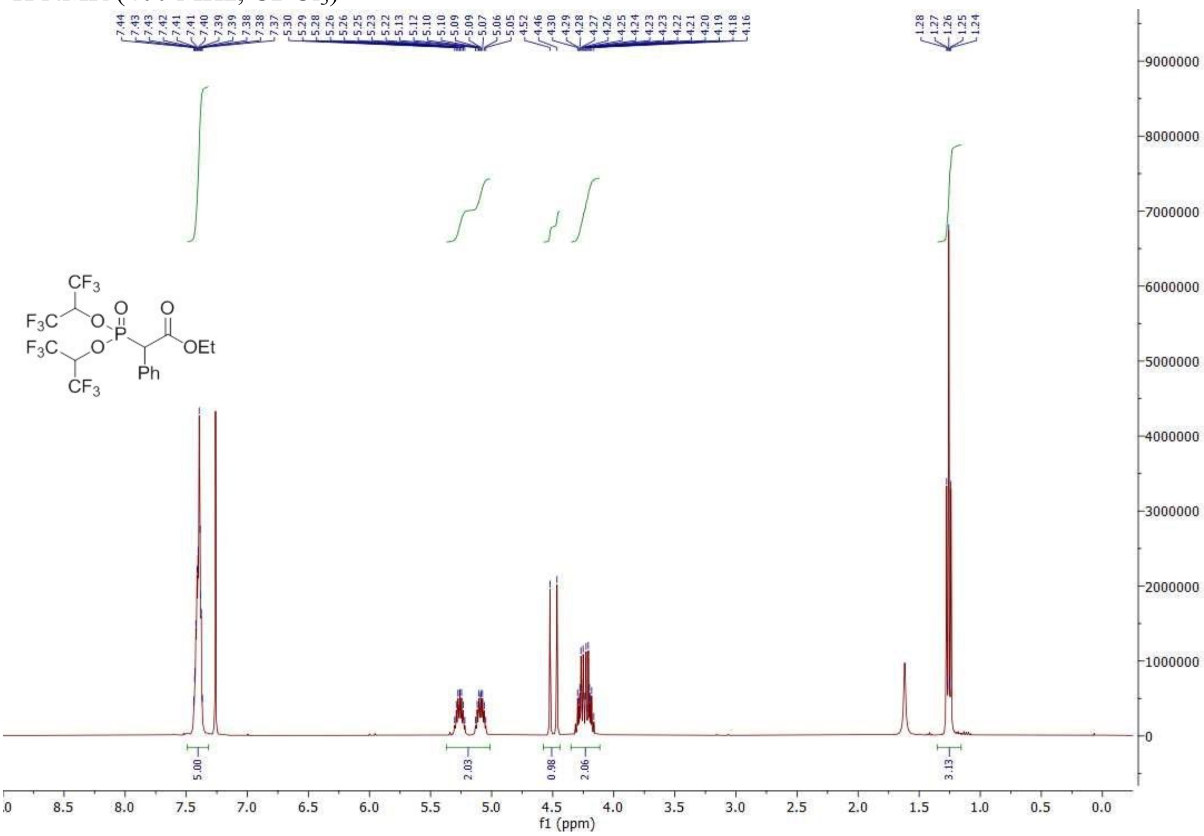

$^{13}C\{^{1}H\}$ NMR (101 MHz, $CDCl_3$)

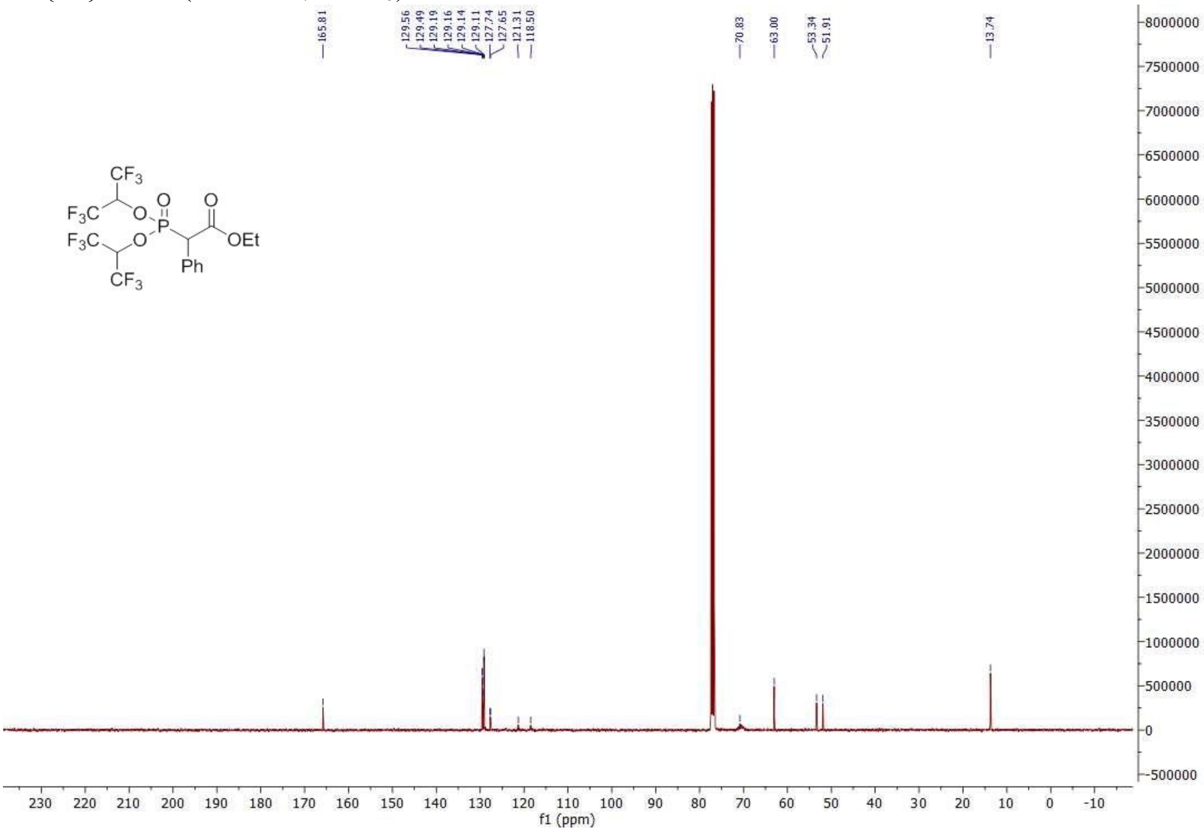

$^{31}P\{^1H\}$ NMR (162 MHz, $CDCl_3$)

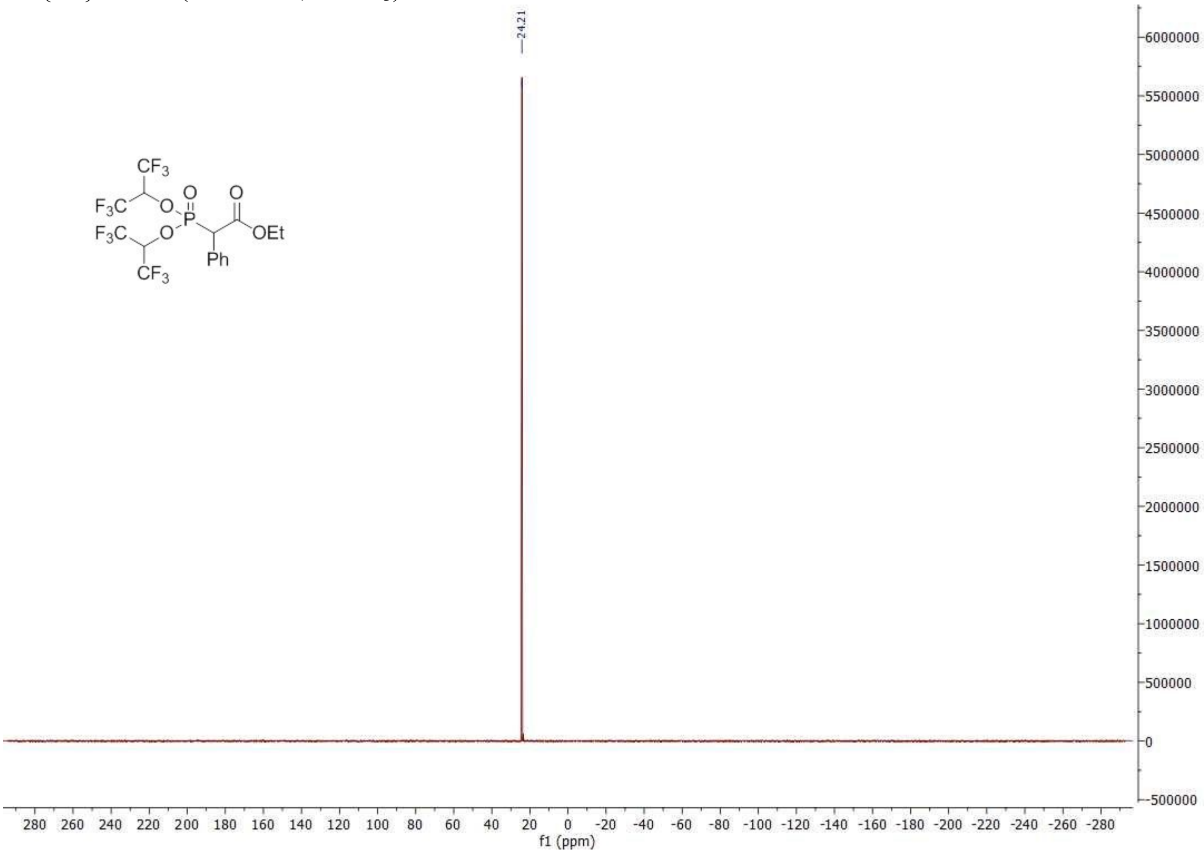

HRMS

Elemental Composition Report **Page 1**

Single Mass Analysis
Tolerance = 5.0 PPM / DBE: min = -1.5, max = 90.0
Element prediction: Off
Number of isotope peaks used for i-FIT = 9

Monoisotopic Mass, Even Electron Ions
244 formula(e) evaluated with 3 results within limits (all results (up to 1000) for each mass)
Elements Used:
C: 0-20 H: 0-20 O: 0-6 F: 0-15 P: 0-1
231120_SG_EA 41 (0.437) Cm (41:56)

TOF MS ES+
3.63e+006

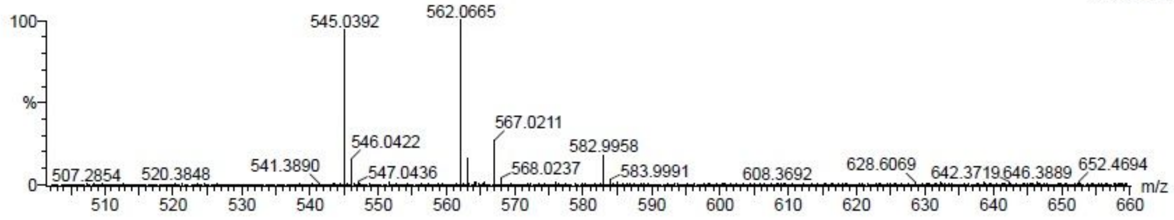

Mass	Calc. Mass	mDa	PPM	DBE	i-FIT	Norm	Conf(%)	Formula
Minimum:				-1.5				
Maximum:		5.0	5.0	90.0				
545.0392	545.0399	-0.7	-1.3	0.5	1106.1	0.186	83.02	C13 H15 O6 F13 P
	545.0387	0.5	0.9	4.5	1107.7	1.805	16.44	C16 H14 O5 F12 P
	545.0376	1.6	2.9	8.5	1111.2	5.220	0.54	C19 H13 O4 F11 P

3a

^{1}H NMR (400 MHz, $CDCl_3$)

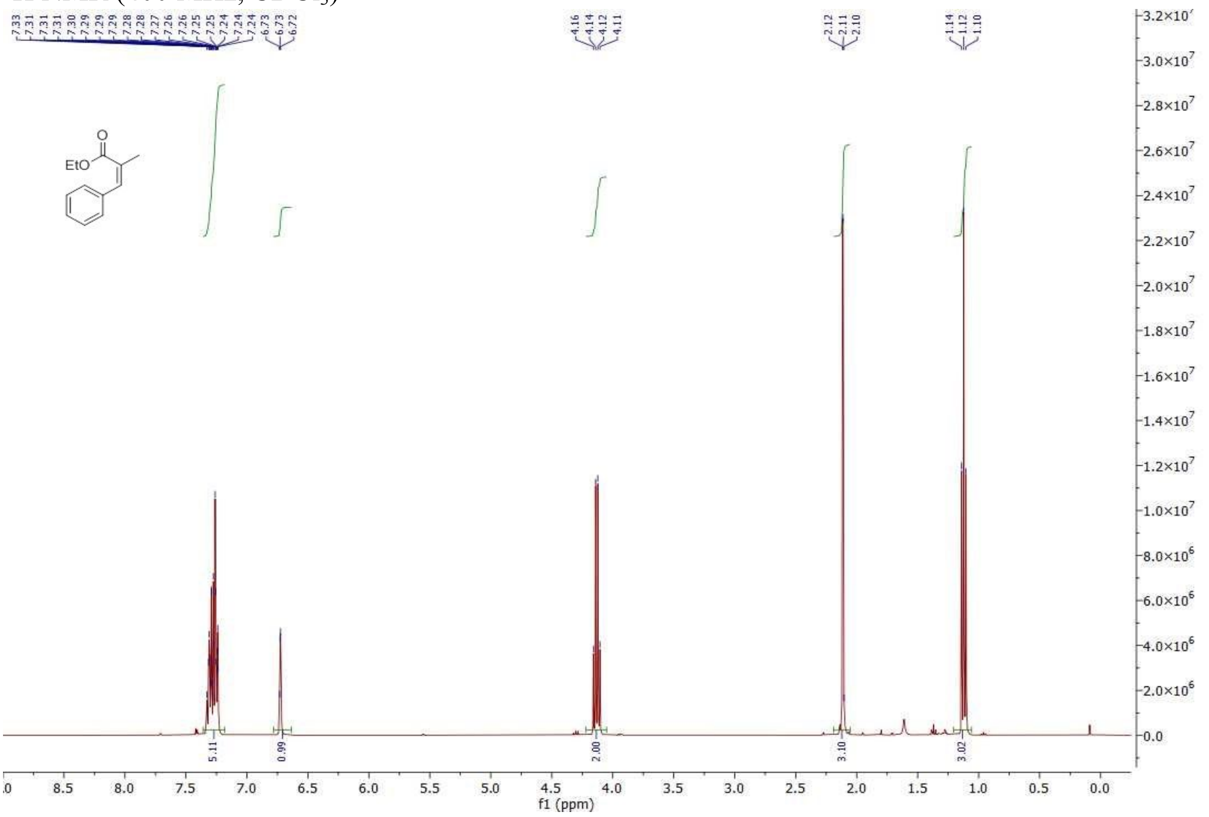

3b

^{1}H NMR (400 MHz, $CDCl_3$)

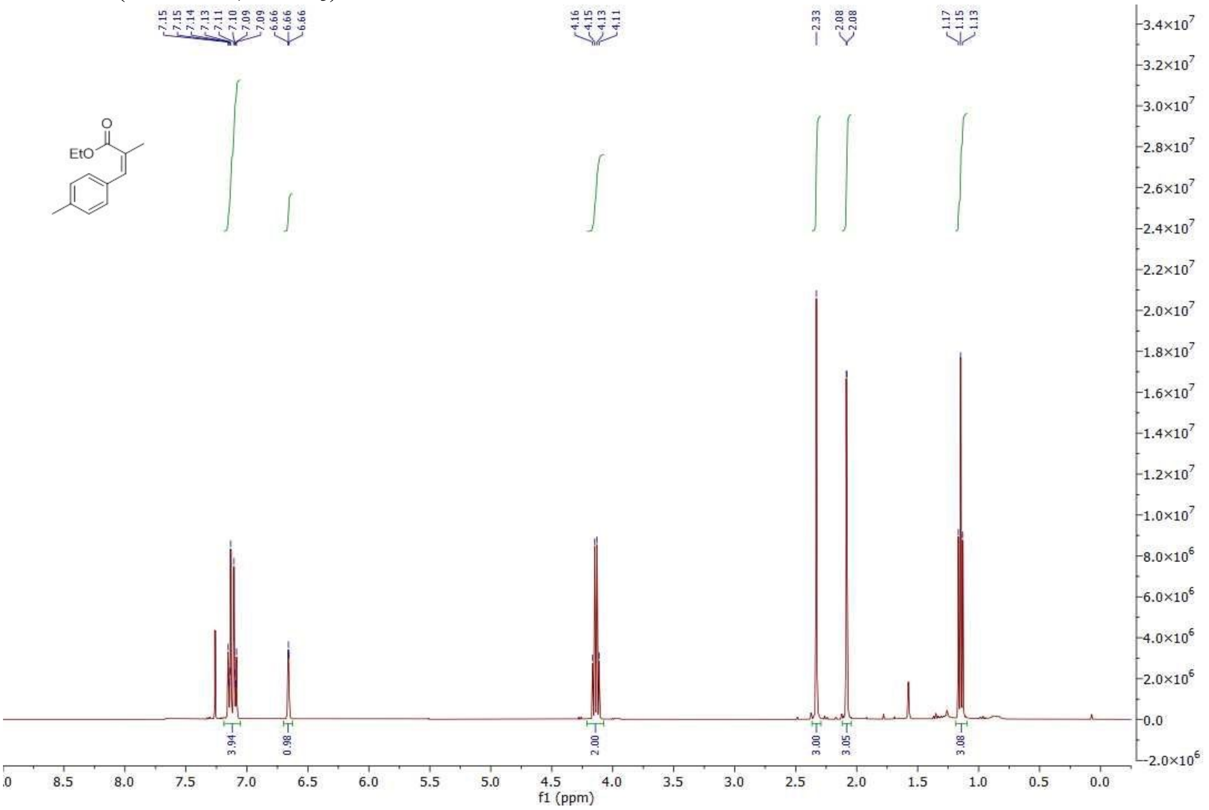

$^{13}C\{^1H\}$ NMR (101 MHz, $CDCl_3$)

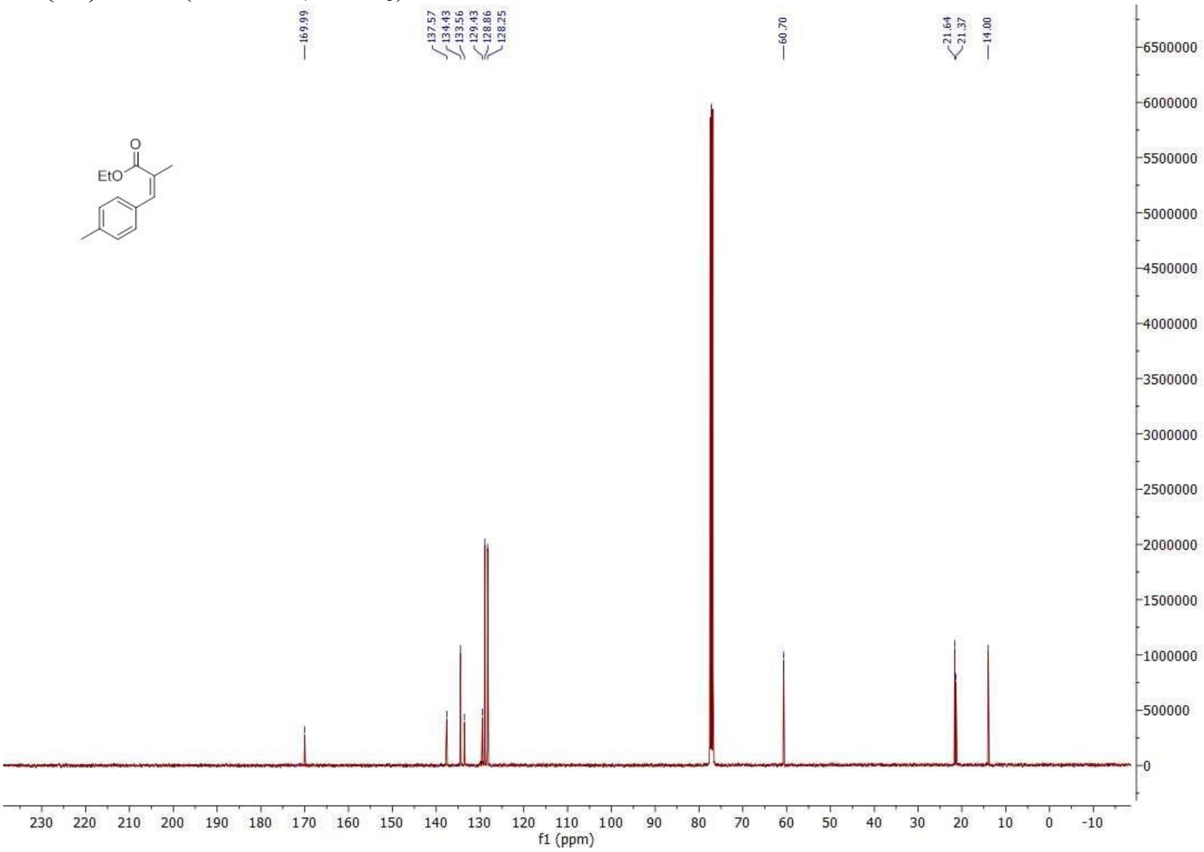

HRMS

Elemental Composition Report **Page 1**

Single Mass Analysis
Tolerance = 5.0 PPM / DBE: min = -1.5, max = 90.0
Element prediction: Off
Number of isotope peaks used for i-FIT = 9

Monoisotopic Mass, Even Electron Ions
61 formula(e) evaluated with 1 results within limits (all results (up to 1000) for each mass)
Elements Used:
C: 0-50 H: 0-50 N: 0-1 O: 0-4 Na: 0-1
230324_SG_32A 12 (0.173) Cm (12:26)

TOF MS ES+
2.67e+006

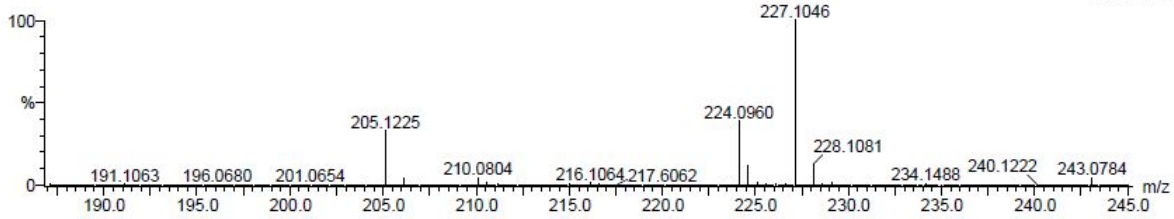

Minimum:				-1.5				
Maximum:		5.0	5.0	90.0				
Mass	Calc. Mass	mDa	PPM	DBE	i-FIT	Norm	Conf(%)	Formula
227.1046	227.1048	-0.2	-0.9	5.5	1685.4	n/a	n/a	C13 H16 O2 Na

3c

^{1}H NMR (400 MHz, $CDCl_3$)

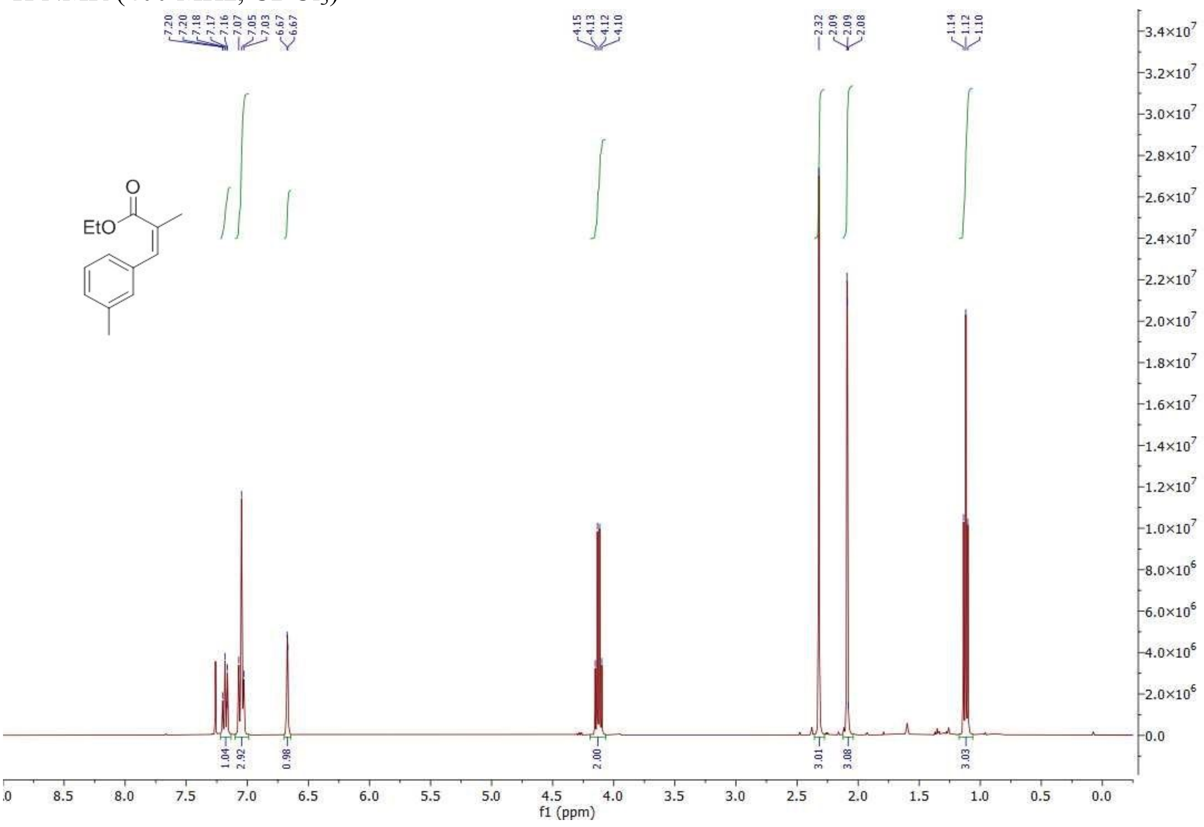

^{13}C{^{1}H} NMR (101 MHz, $CDCl_3$)

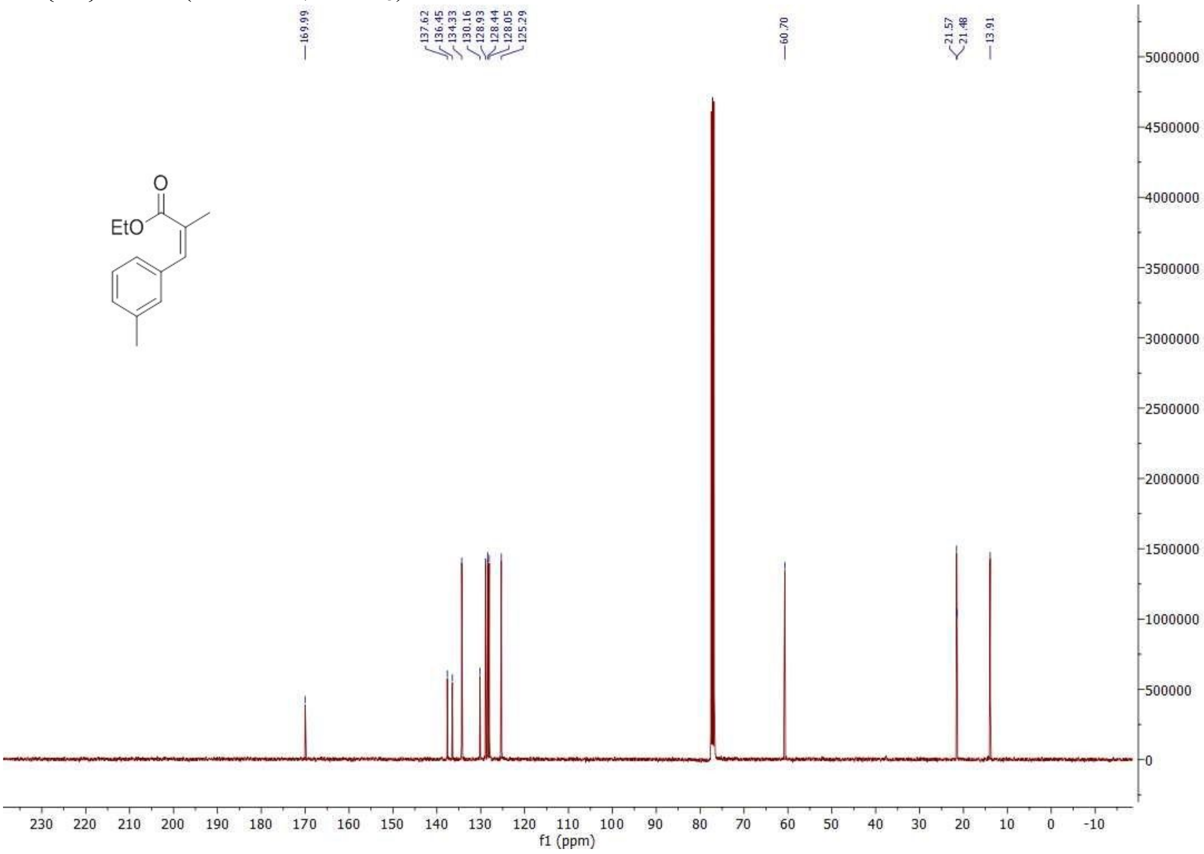

3d

^{1}H NMR (400 MHz, $CDCl_3$)

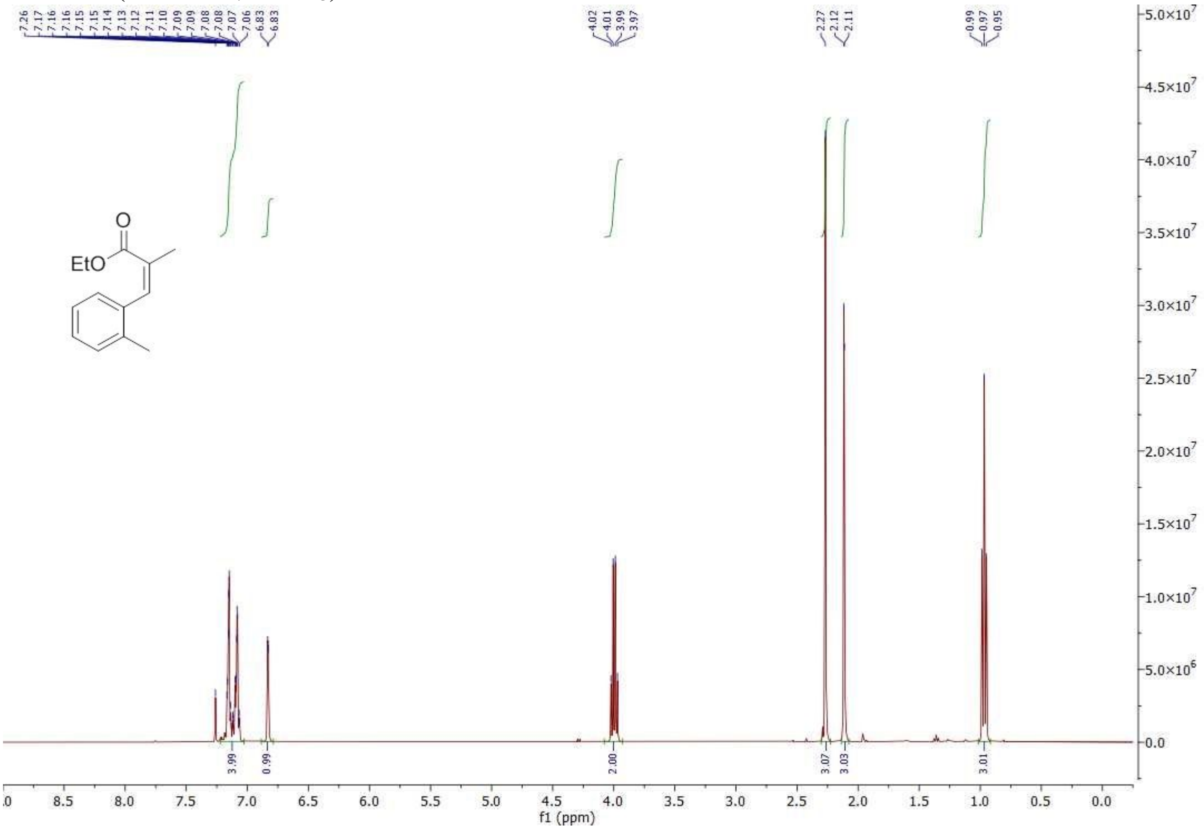

$^{13}C\{^{1}H\}$ NMR (101 MHz, $CDCl_3$)

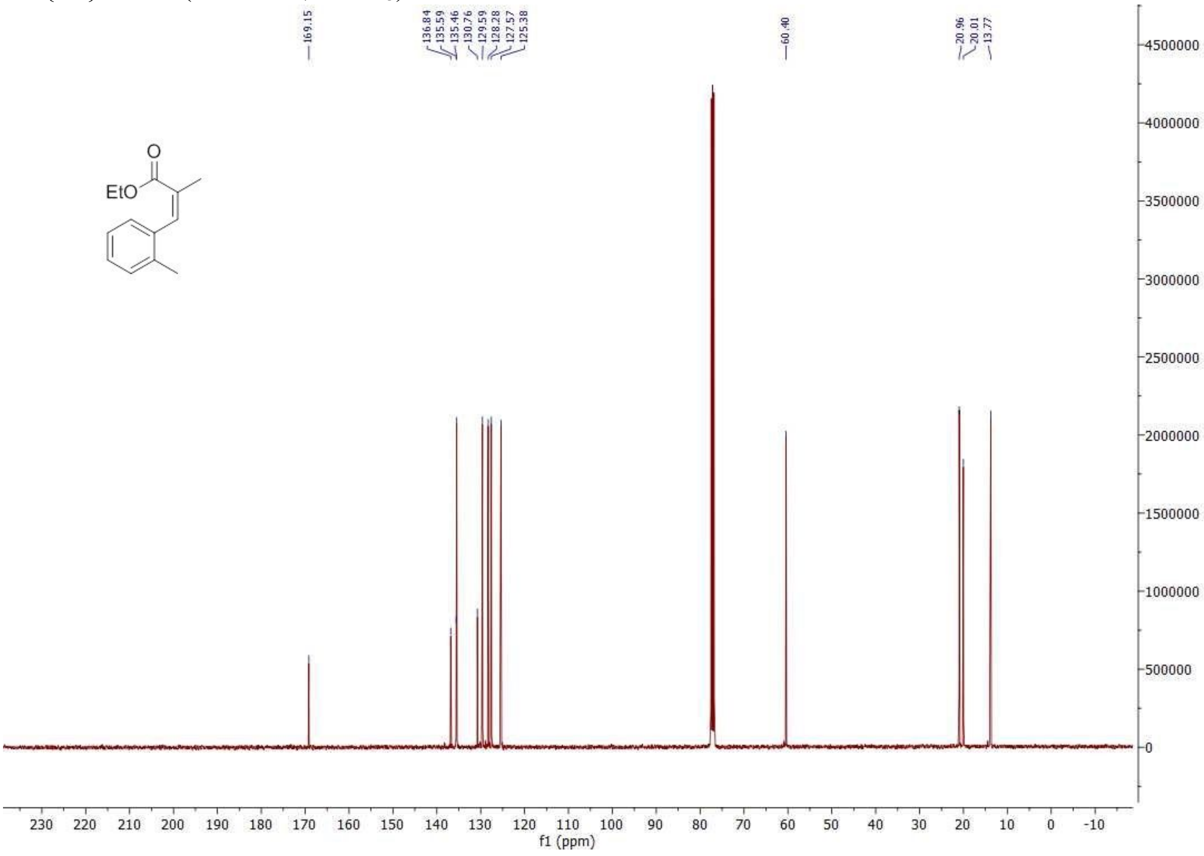

HRMS

Elemental Composition Report Page 1

Single Mass Analysis
Tolerance = 5.0 PPM / DBE: min = -1.5, max = 90.0
Element prediction: Off
Number of isotope peaks used for i-FIT = 9

Monoisotopic Mass, Even Electron Ions
12 formula(e) evaluated with 1 results within limits (all results (up to 1000) for each mass)
Elements Used:
C: 0-25 H: 0-25 O: 0-4
230406_SG_33_apciA 8 (0.211) Cm (8:14-2:5)

TOF MS AP+
8.35e+004

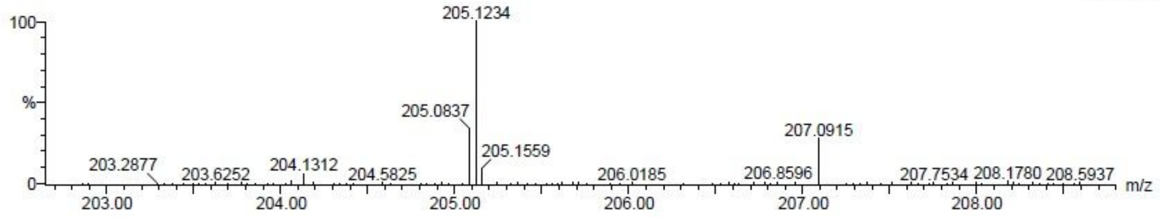

Mass	Calc. Mass	mDa	PPM	DBE	i-FIT	Norm	Conf(%)	Formula
Minimum:				-1.5				
Maximum:		5.0	5.0	90.0				
205.1234	205.1229	0.5	2.4	5.5	528.4	n/a	n/a	C13 H17 O2

3e

^{1}H NMR (400 MHz, $CDCl_3$)

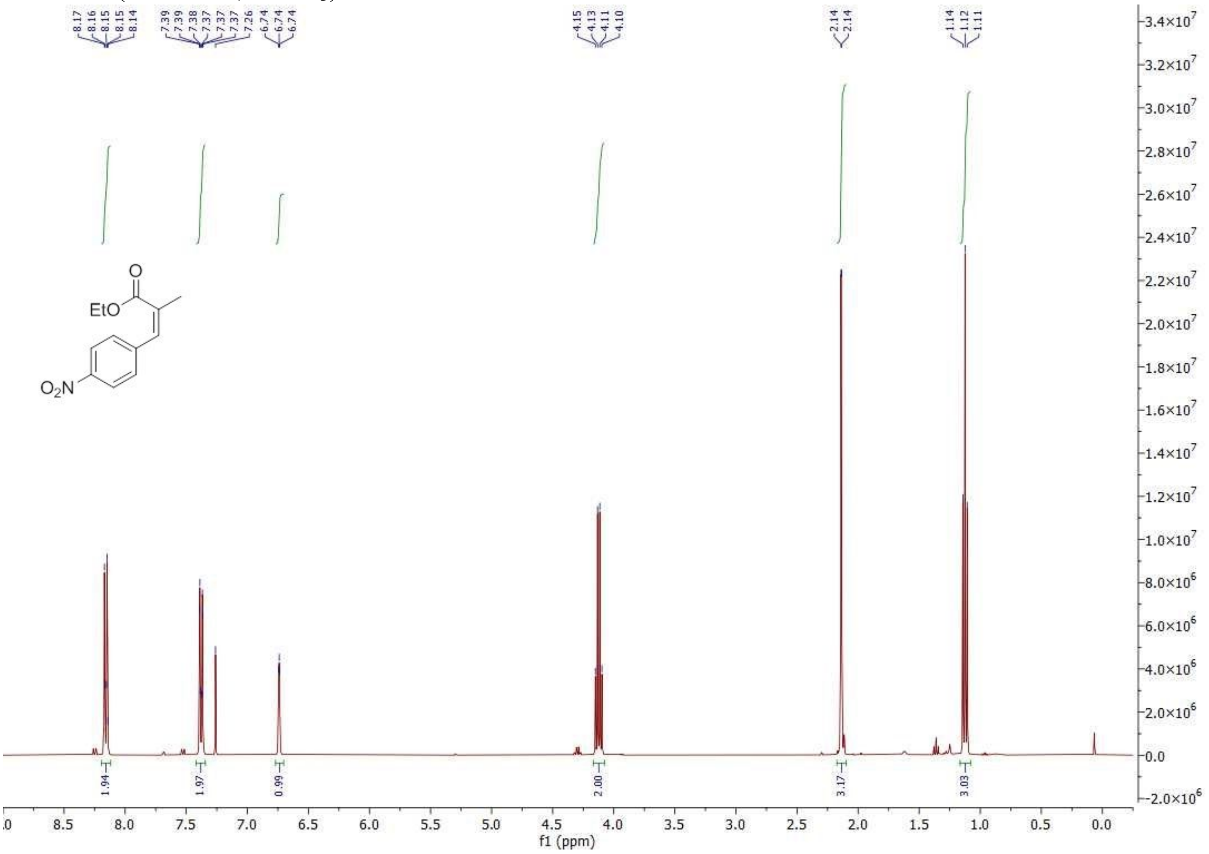

$^{13}C\{^{1}H\}$ NMR (101 MHz, $CDCl_3$)

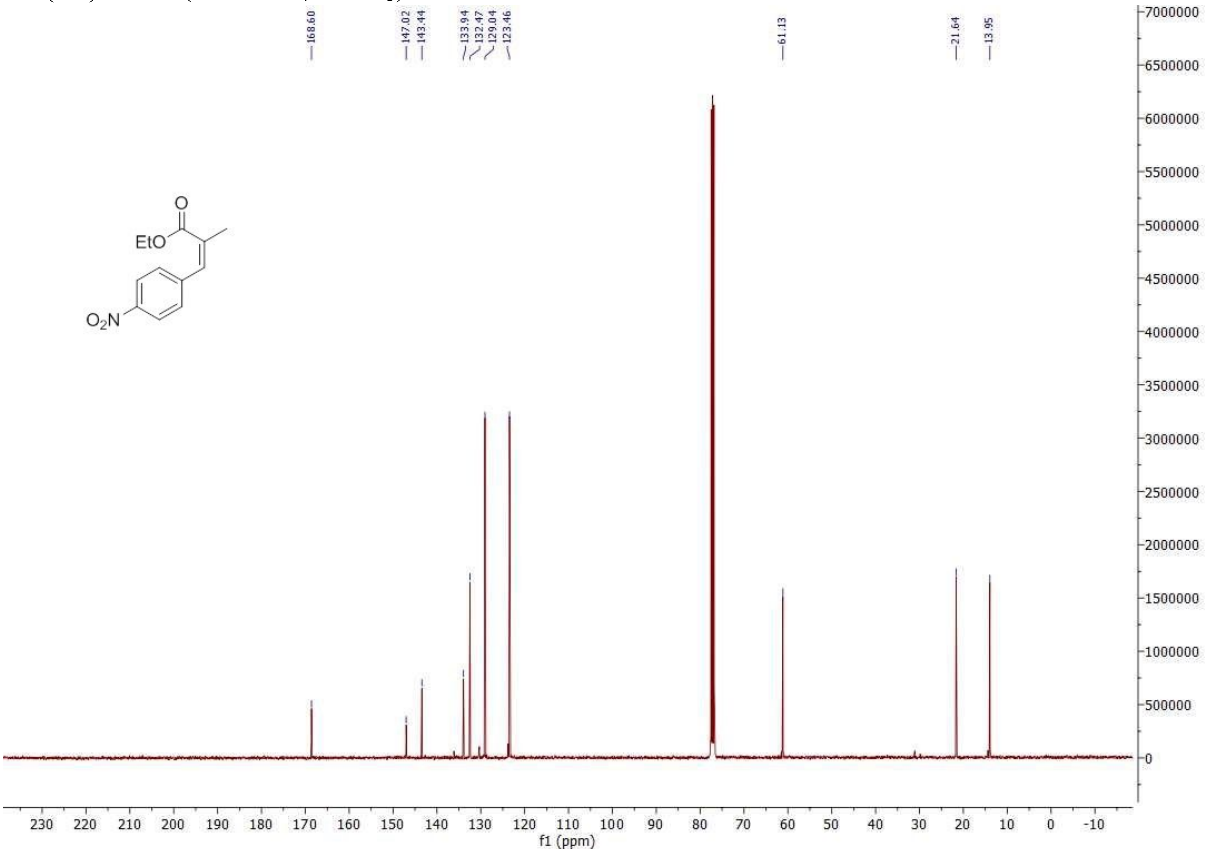

HRMS

Elemental Composition Report **Page 1**

Single Mass Analysis
Tolerance = 5.0 PPM / DBE: min = -1.5, max = 90.0
Element prediction: Off
Number of isotope peaks used for i-FIT = 9

Monoisotopic Mass, Even Electron Ions
381 formula(e) evaluated with 2 results within limits (all results (up to 1000) for each mass)
Elements Used:
C: 0-30 H: 0-40 N: 0-6 O: 0-13 Na: 0-1
230324_SG_31_apci 8 (0.211) Cm (8:9-21:28)

1: TOF MS AP+
6.64e+005

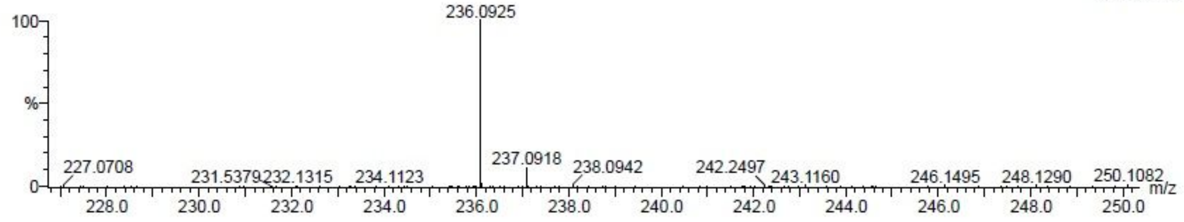

Minimum:				-1.5				
Maximum:		5.0	5.0	90.0				
Mass	Calc. Mass	mDa	PPM	DBE	i-FIT	Norm	Conf(%)	Formula
236.0925	236.0923	0.2	0.8	6.5	338.5	0.759	46.79	C12 H14 N O4
	236.0936	-1.1	-4.7	11.5	338.3	0.631	53.21	C13 H10 N5

3f

^{1}H NMR (400 MHz, $CDCl_3$)

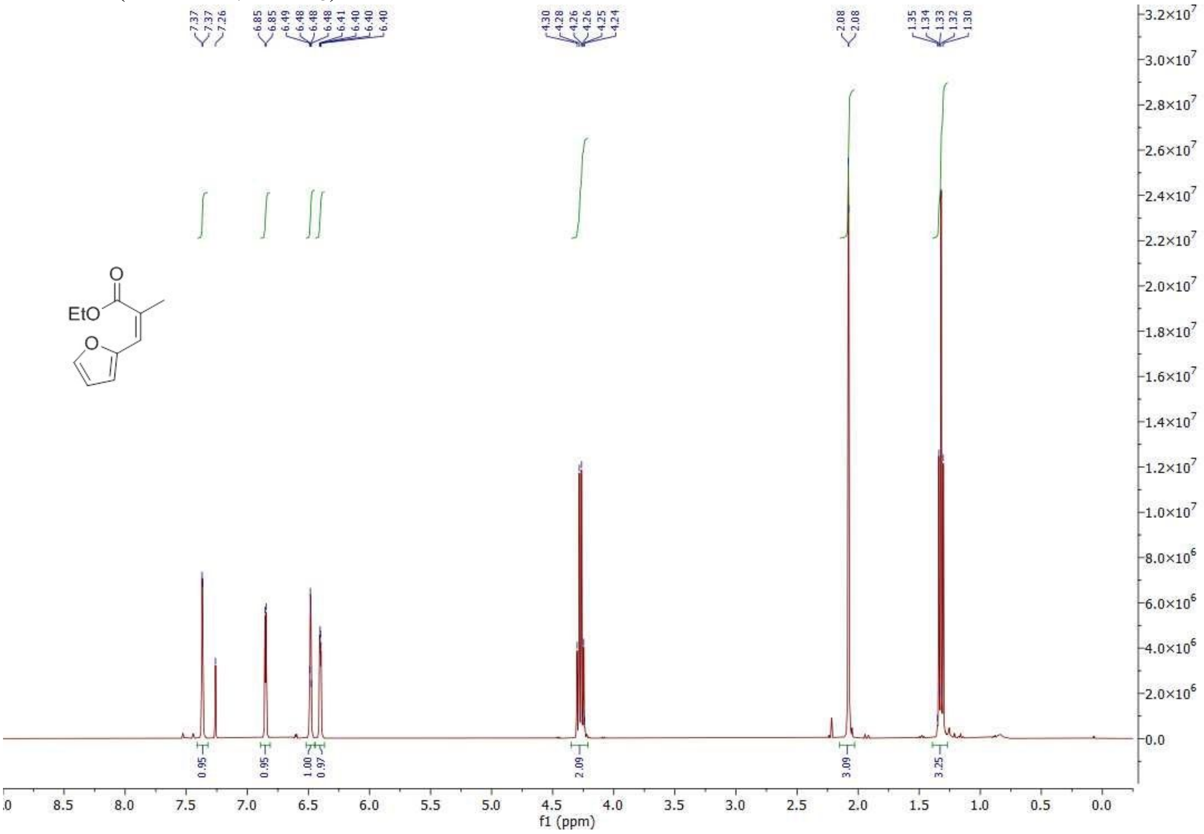

3g

^{1}H NMR (400 MHz, $CDCl_3$)

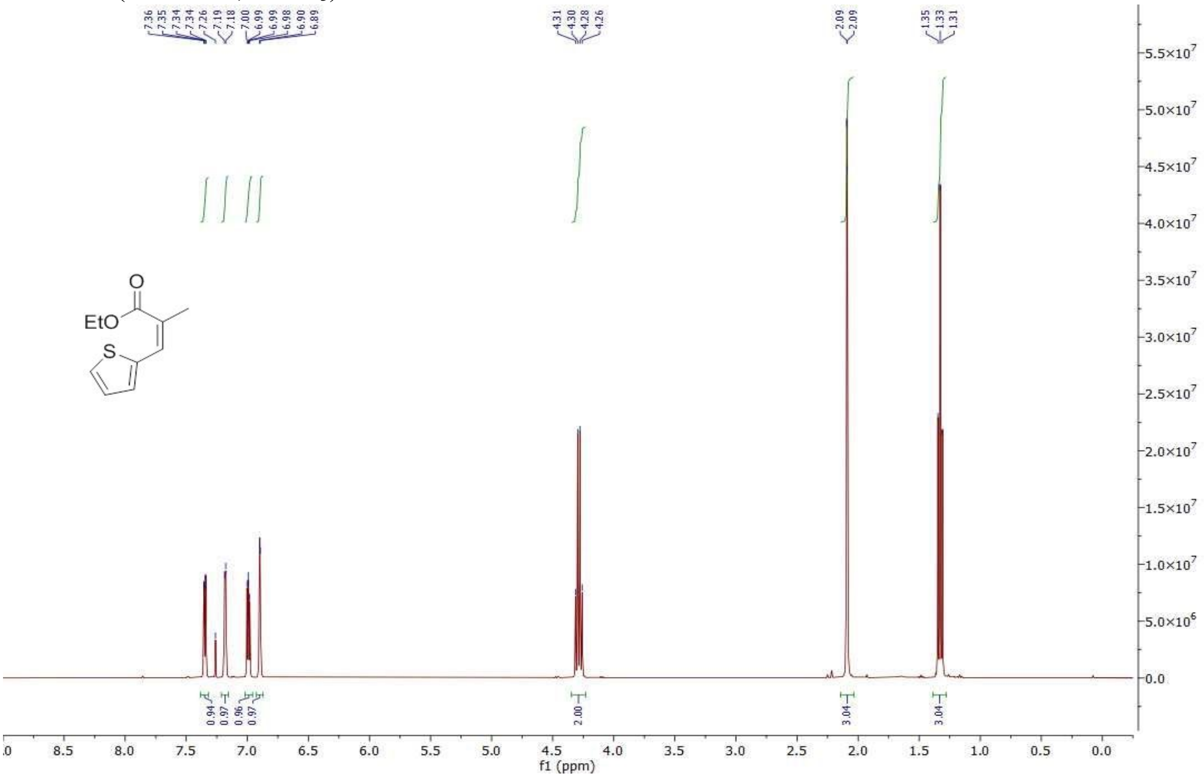

$^{13}C\{^{1}H\}$ NMR (101 MHz, $CDCl_3$)

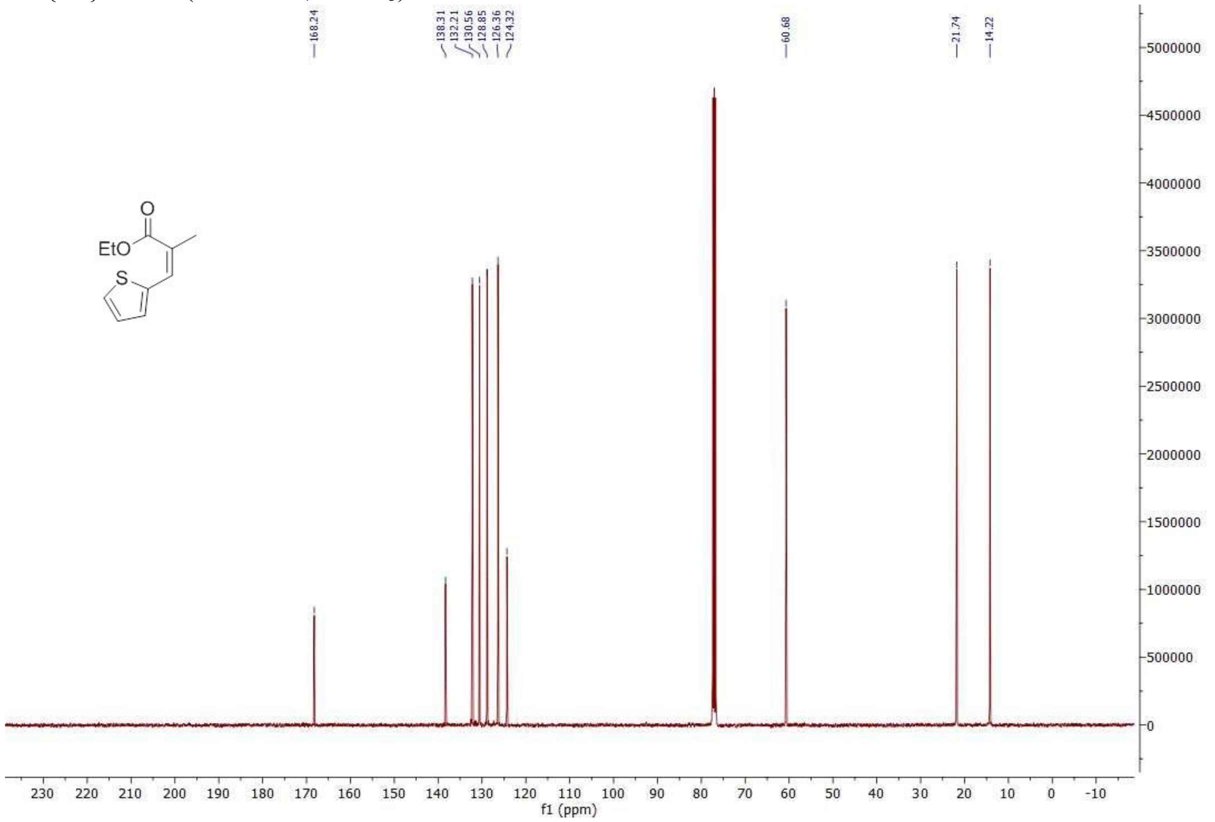

3h

^{1}H NMR (400 MHz, $CDCl_3$)

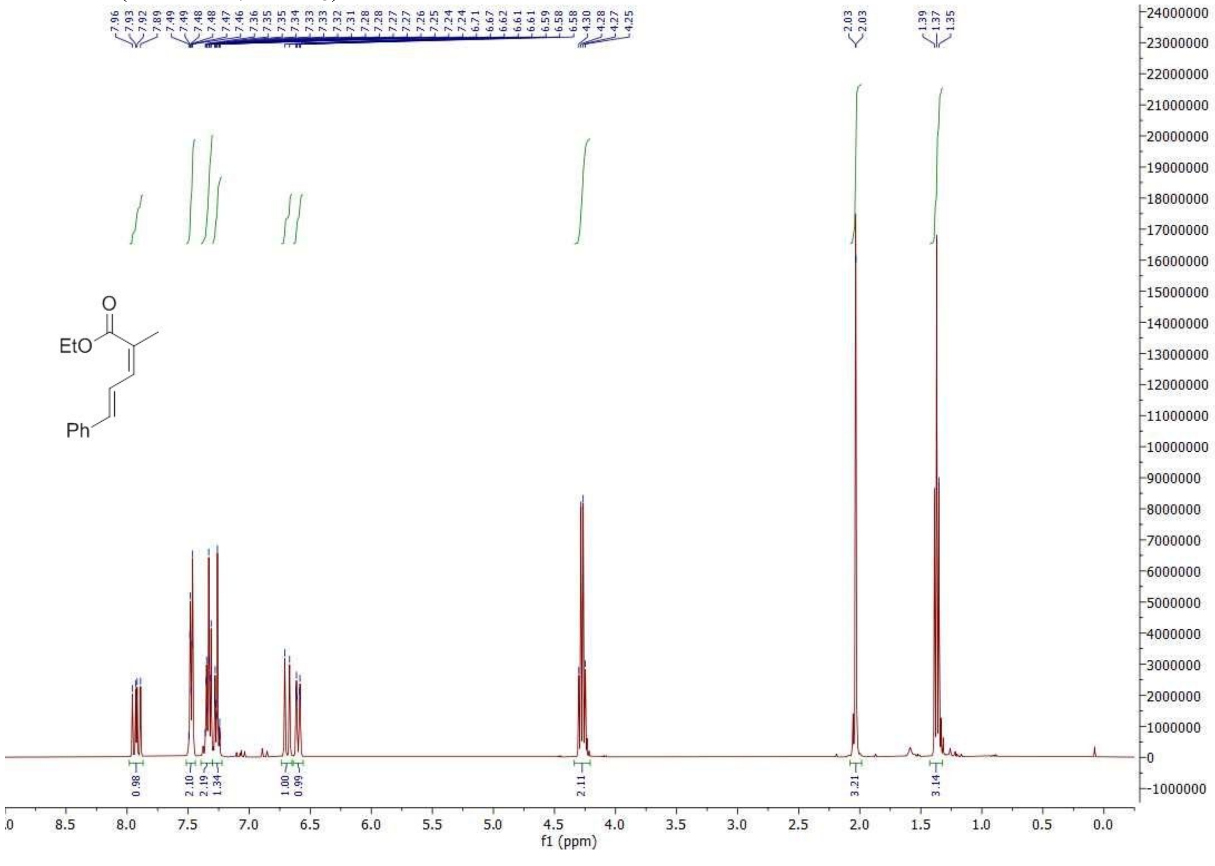

3i

^{1}H NMR (400 MHz, $CDCl_3$)

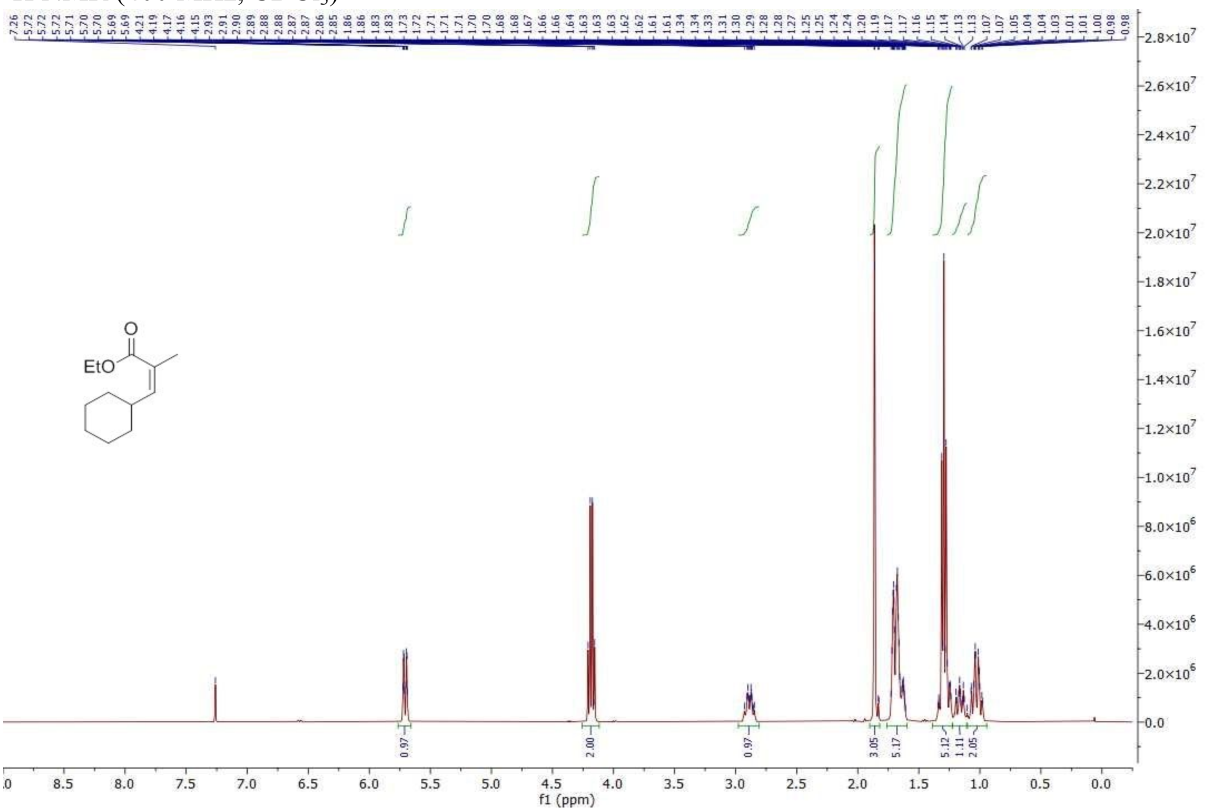

3j

^{1}H NMR (400 MHz, $CDCl_3$)

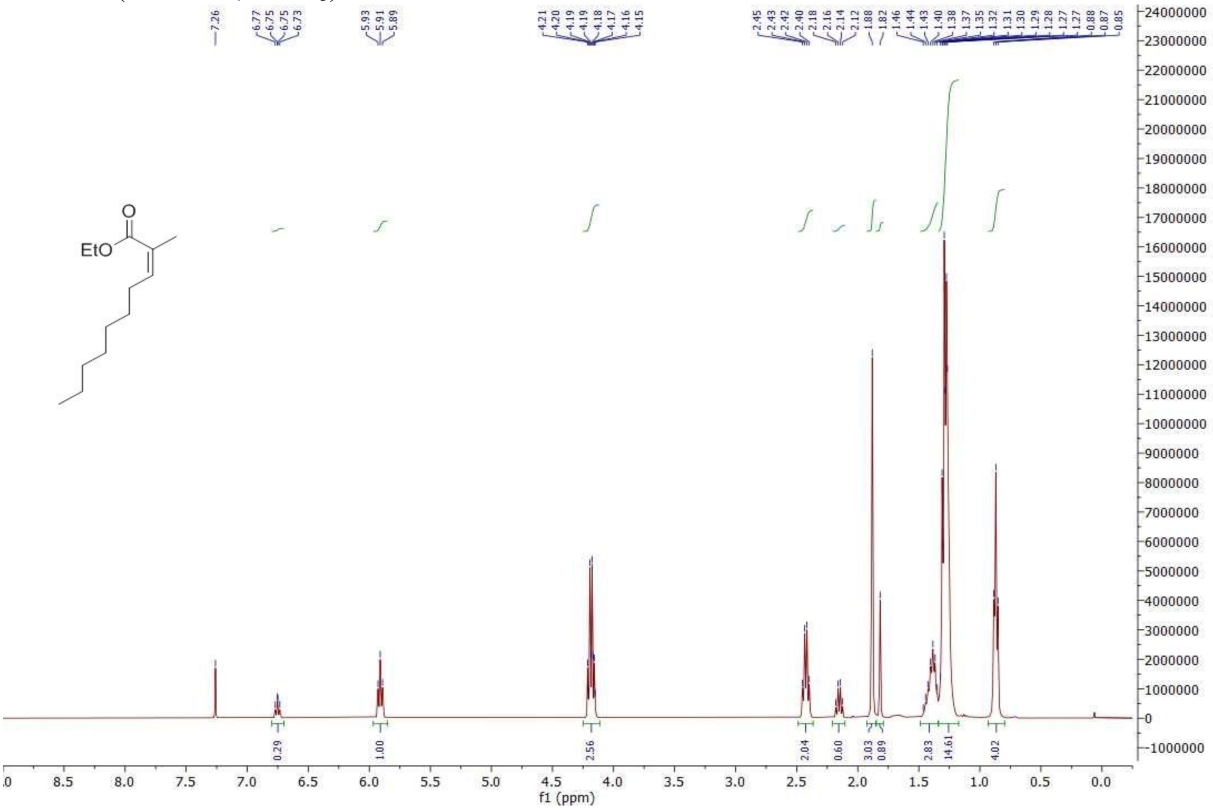

3k

^{1}H NMR (400 MHz, $CDCl_3$)

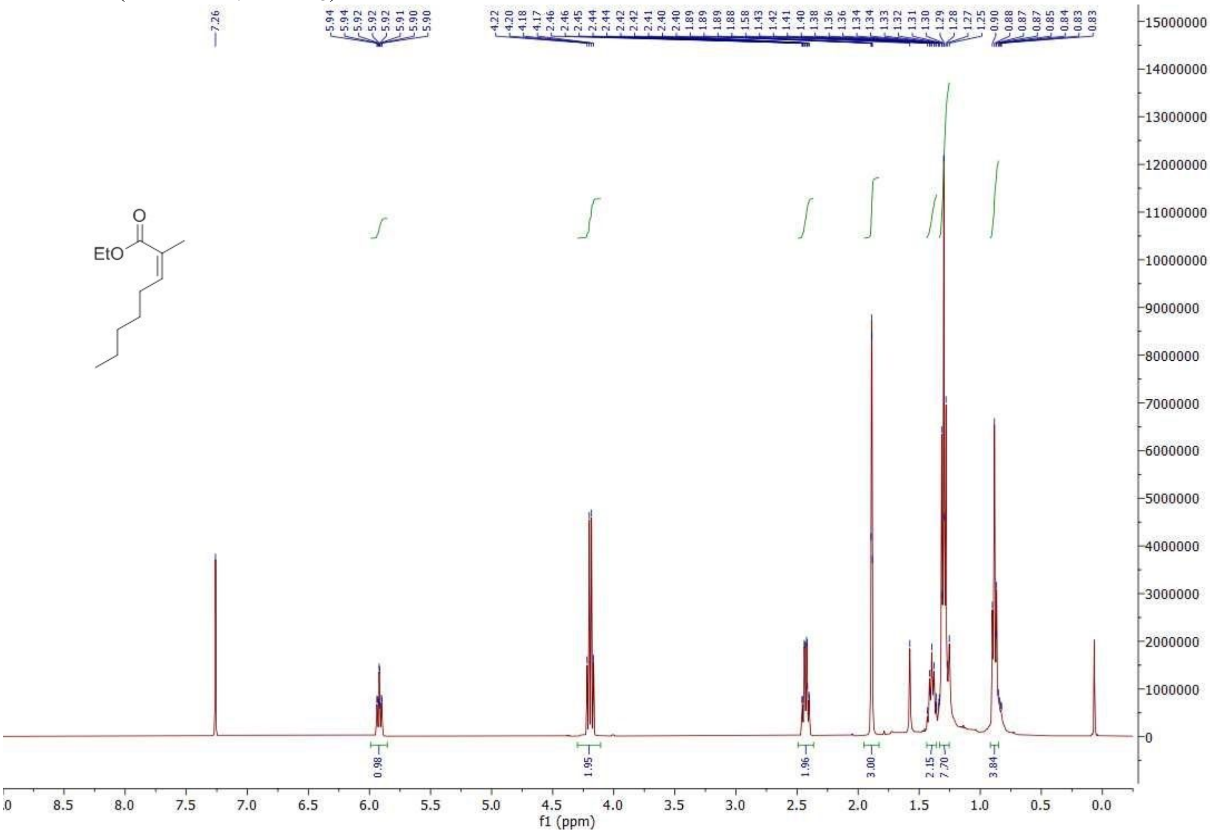

3l

^{1}H NMR (400 MHz, $CDCl_3$)

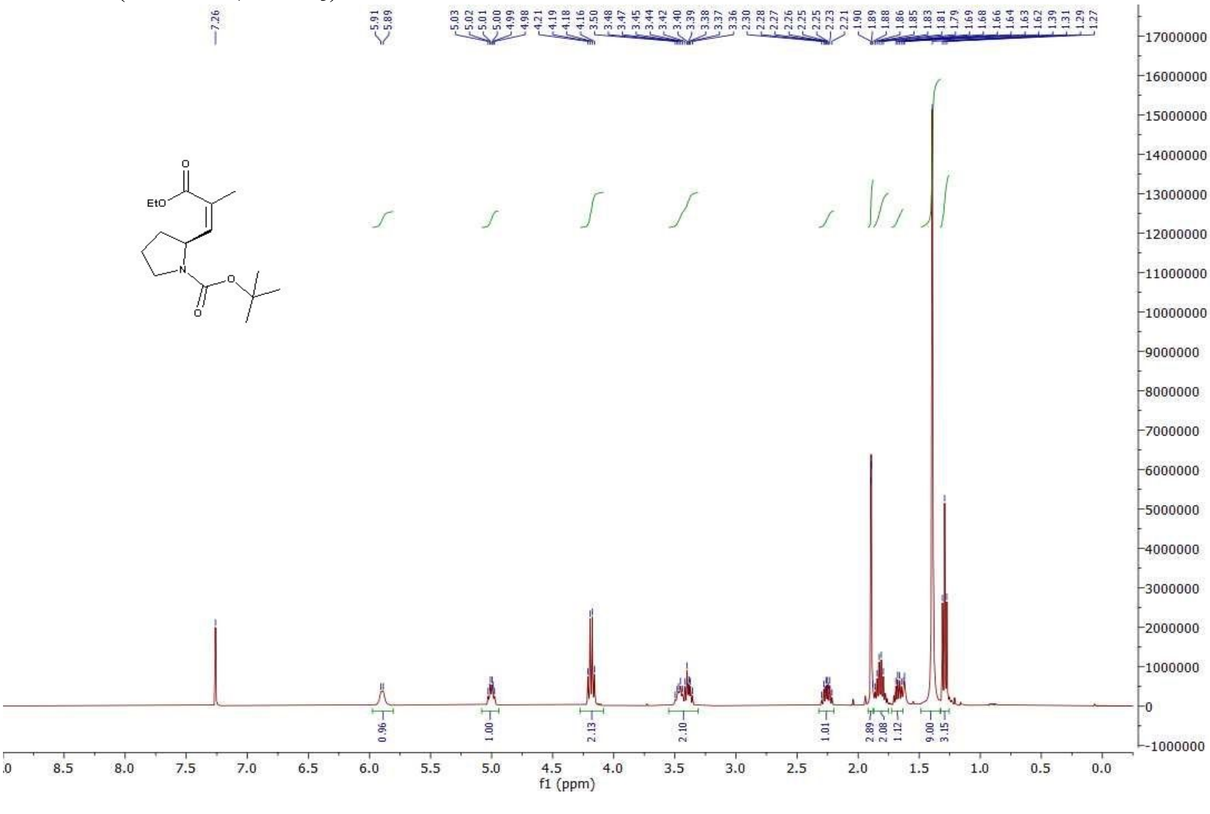

4a

^{1}H NMR (400 MHz, $CDCl_3$)

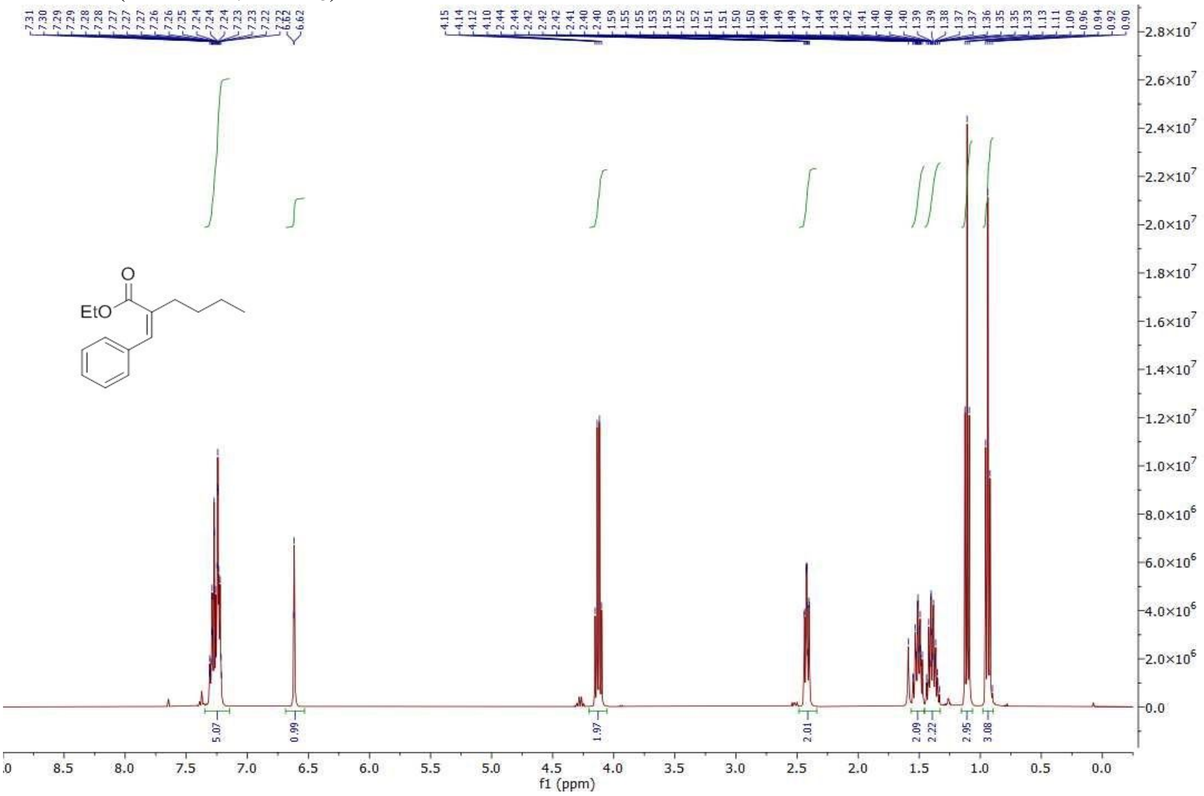

^{13}C{^{1}H} NMR (101 MHz, $CDCl_3$)

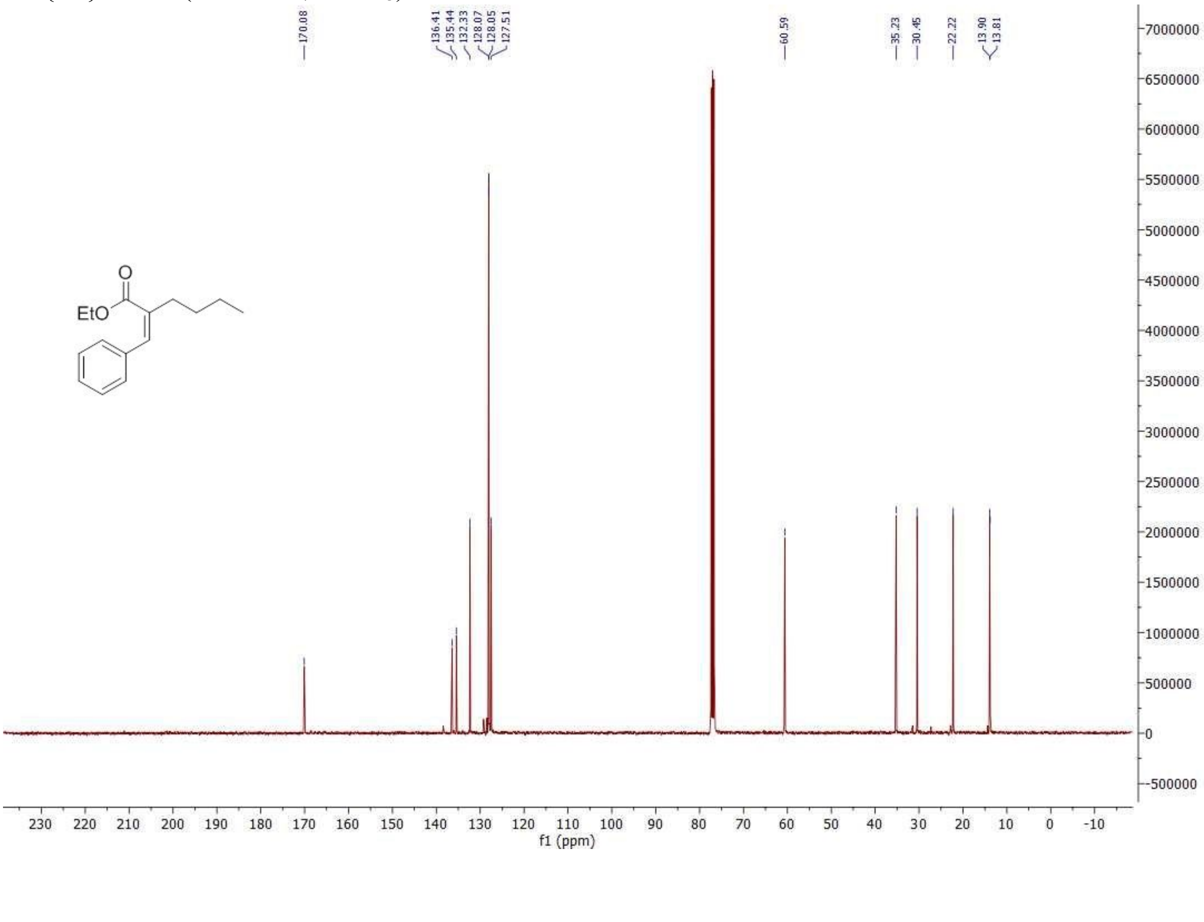

NOESY spectra

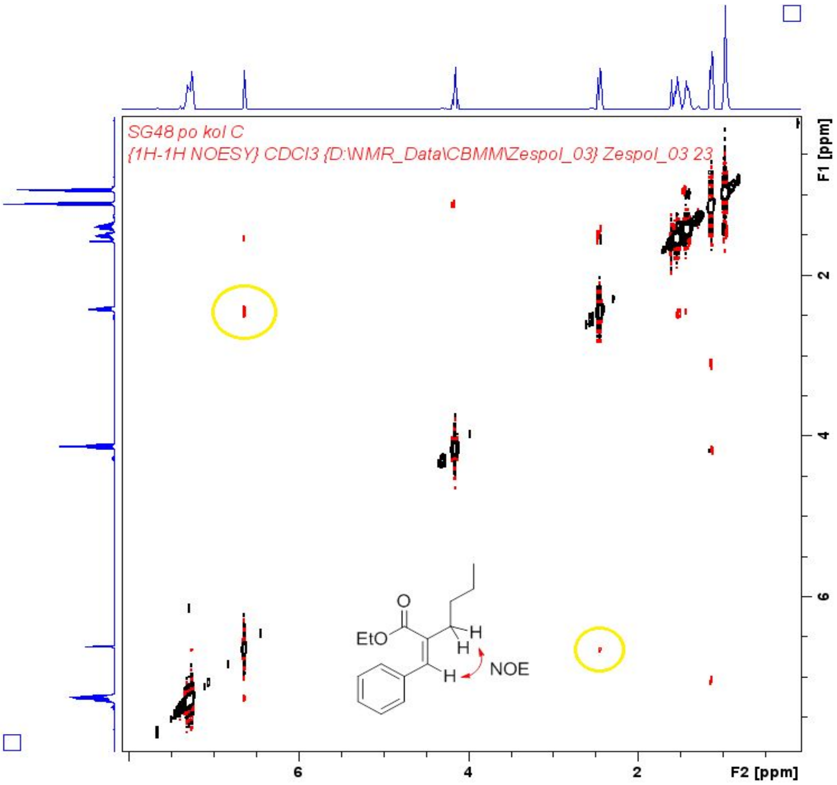

4b

^1H NMR (400 MHz, $CDCl_3$)

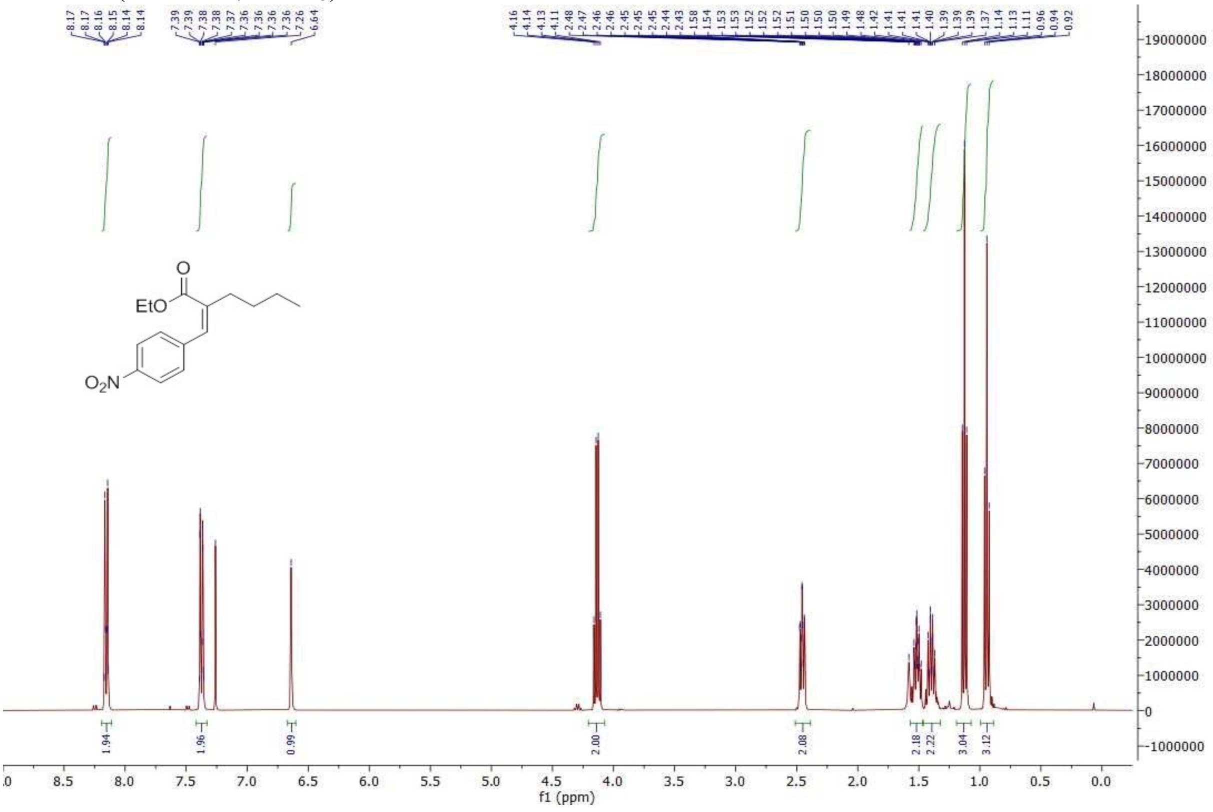

$^{13}C\{^{1}H\}$ NMR (101 MHz, $CDCl_3$)

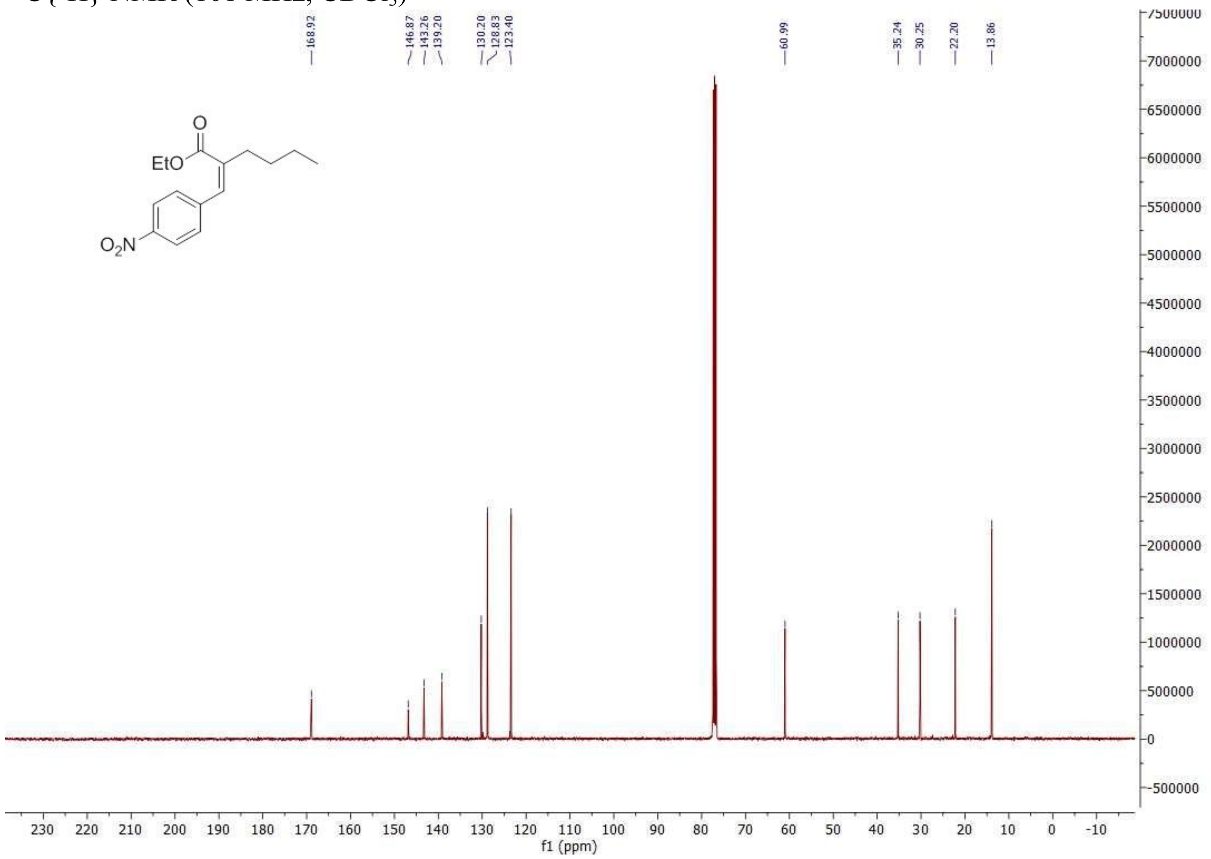

NOESY spectra

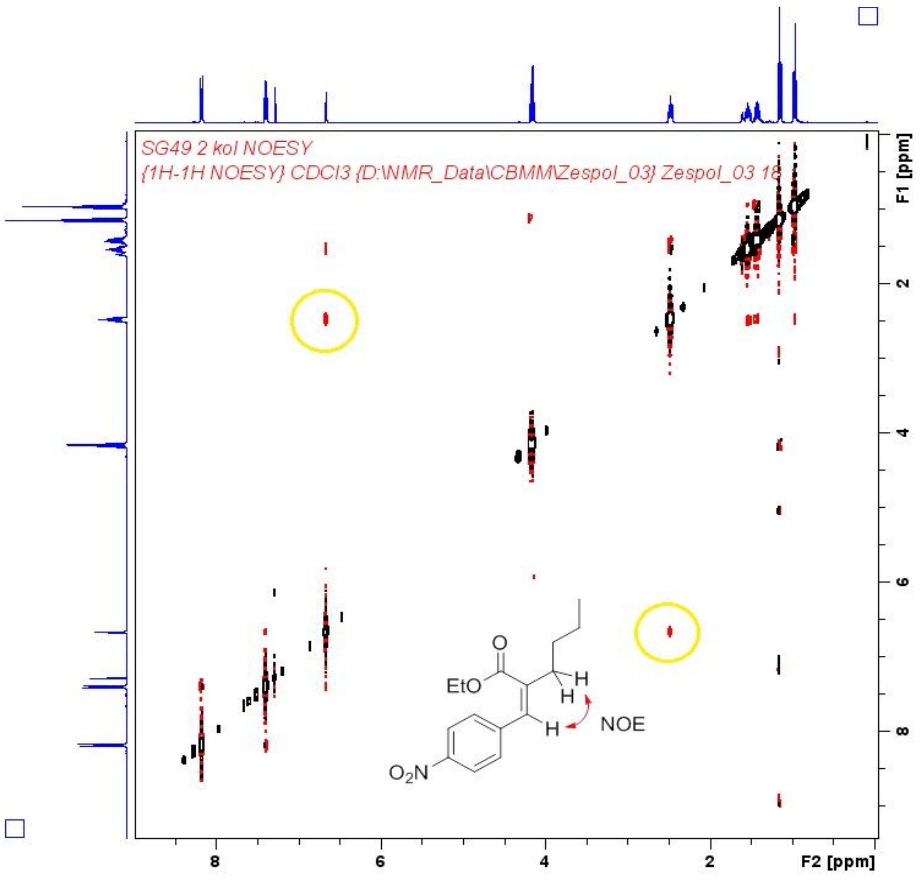

4c

^{1}H NMR (400 MHz, $CDCl_3$)

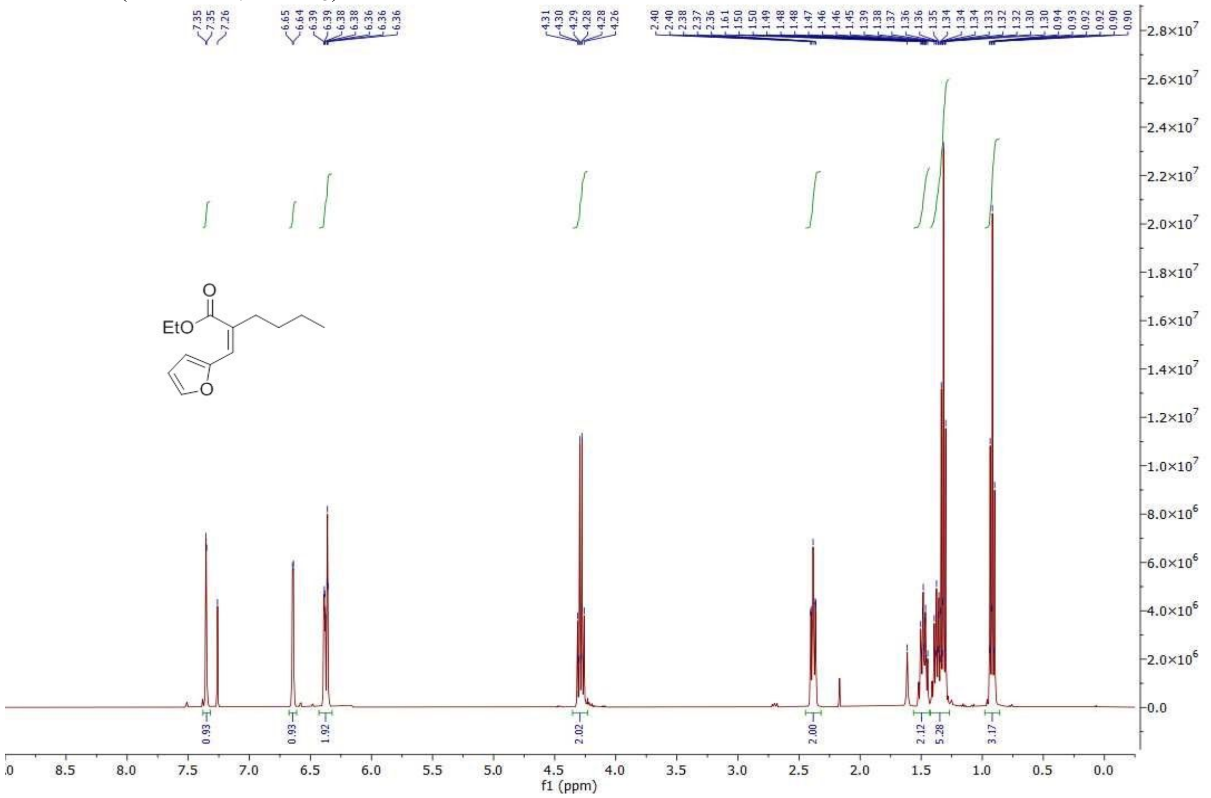

^{13}C{^{1}H} NMR (101 MHz, $CDCl_3$)

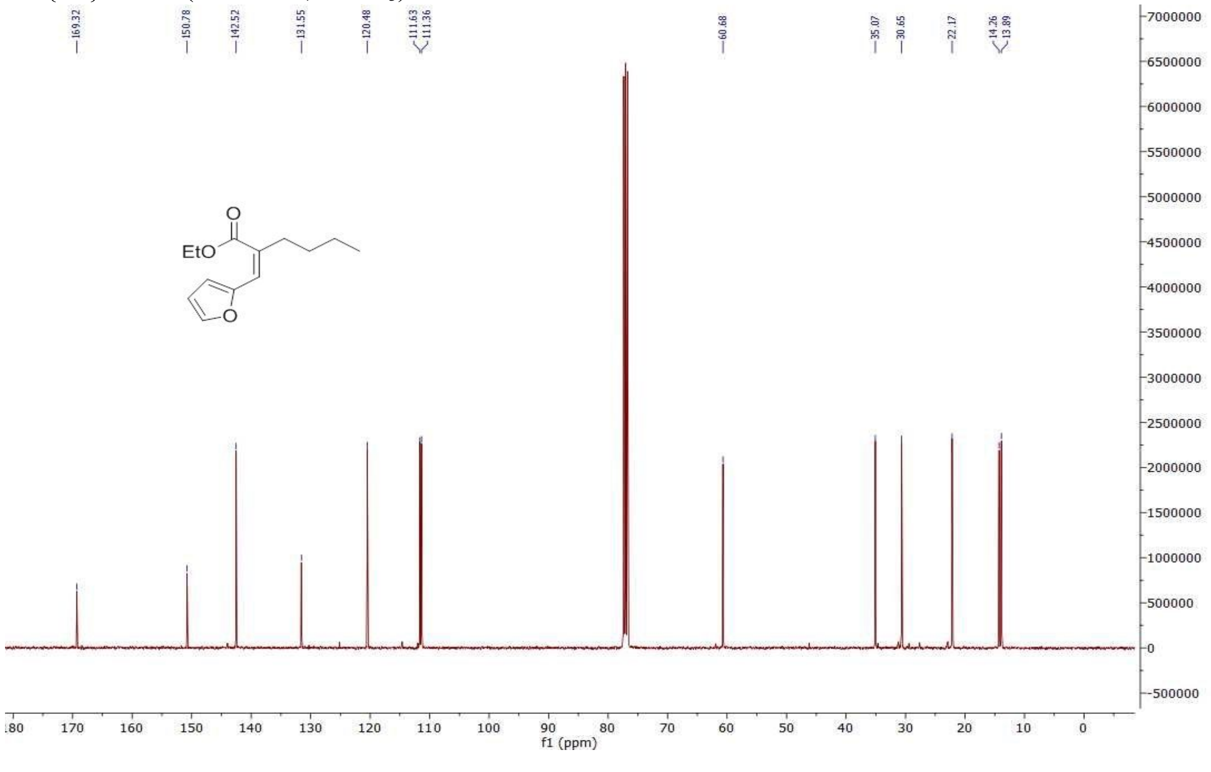

NOESY spectra

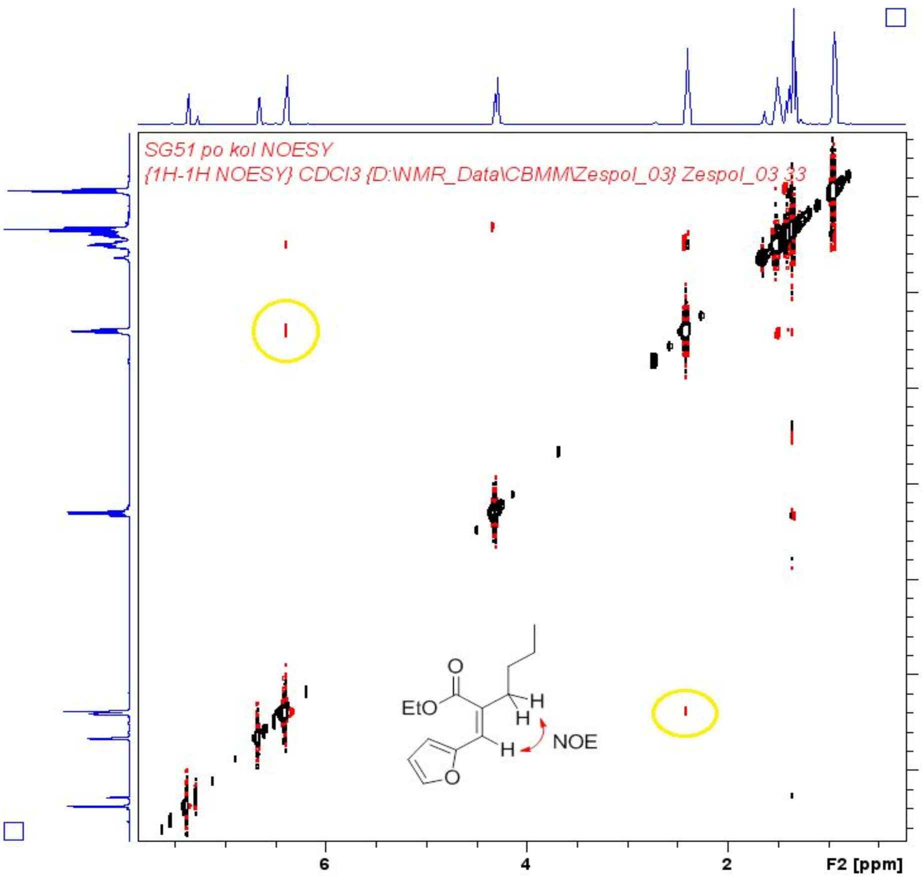

4d

^{1}H NMR (400 MHz, $CDCl_3$)

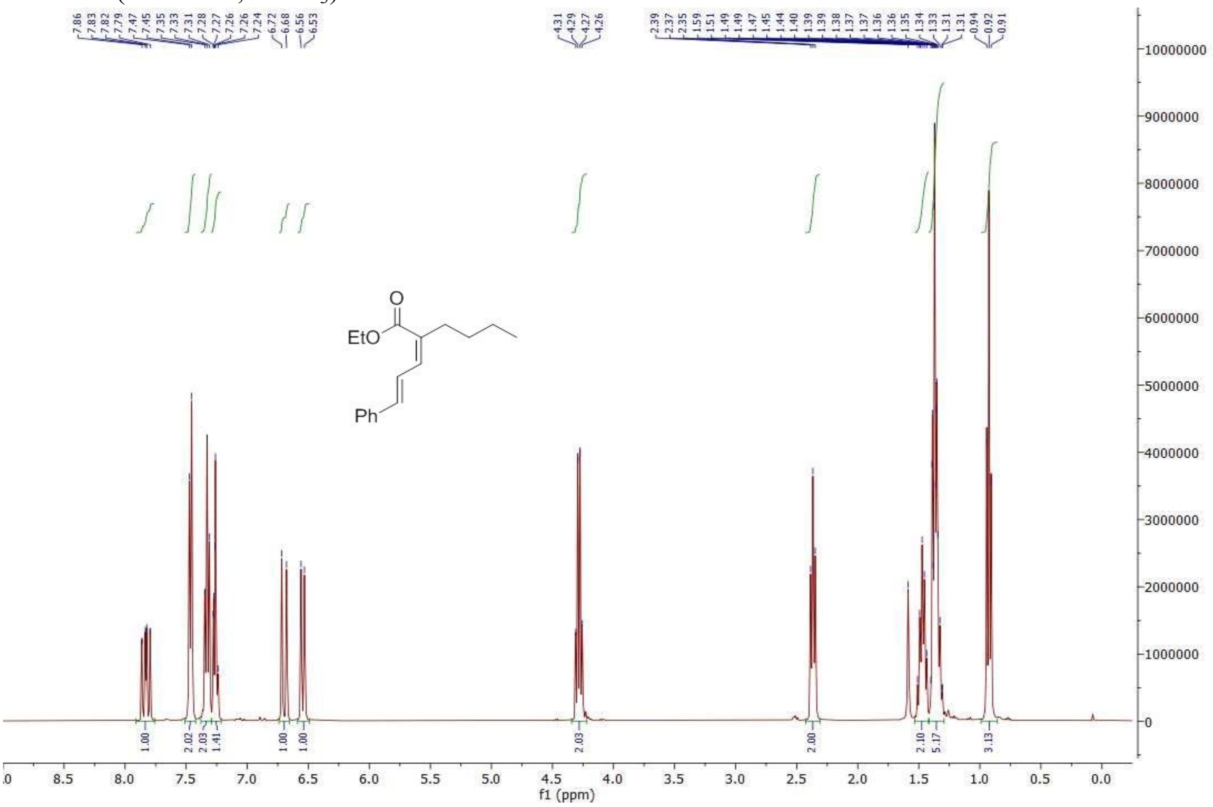

$^{13}C\{^{1}H\}$ NMR (101 MHz, $CDCl_3$)

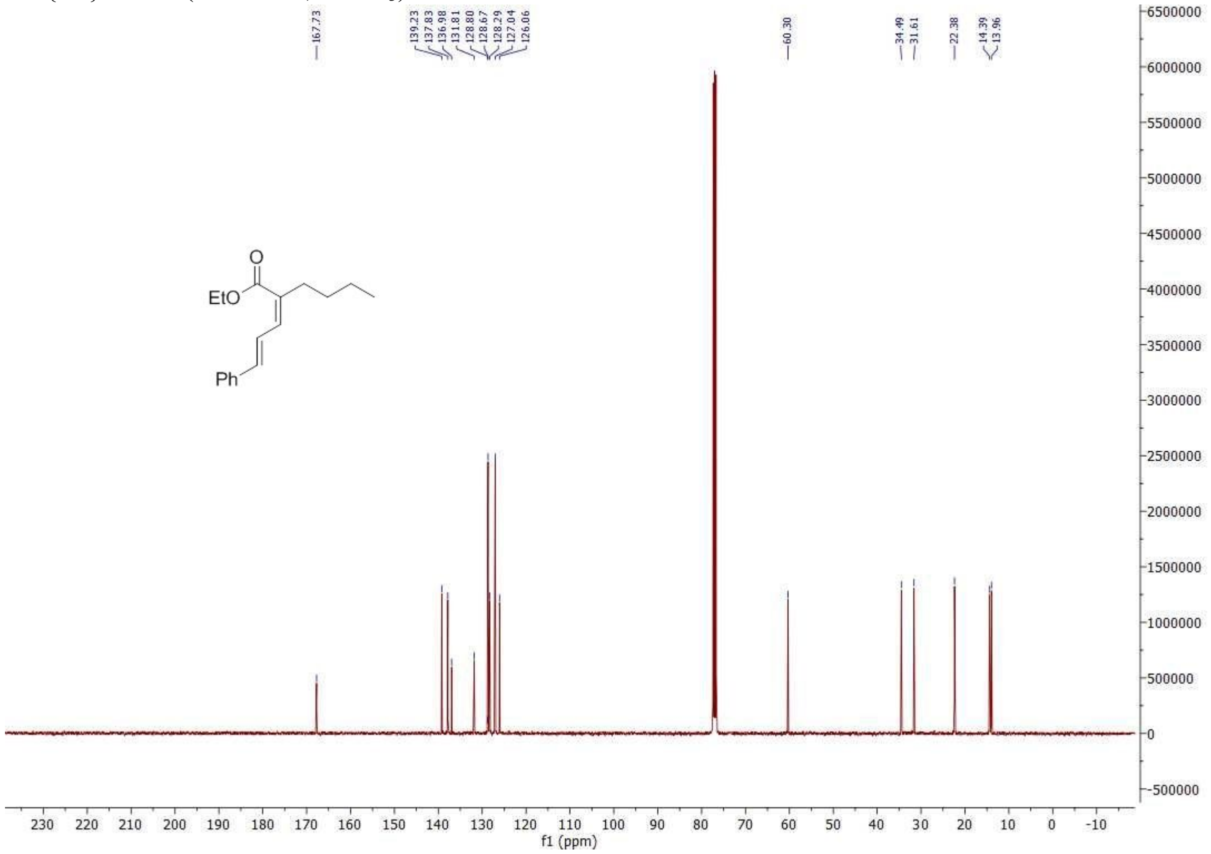

NOESY spectra

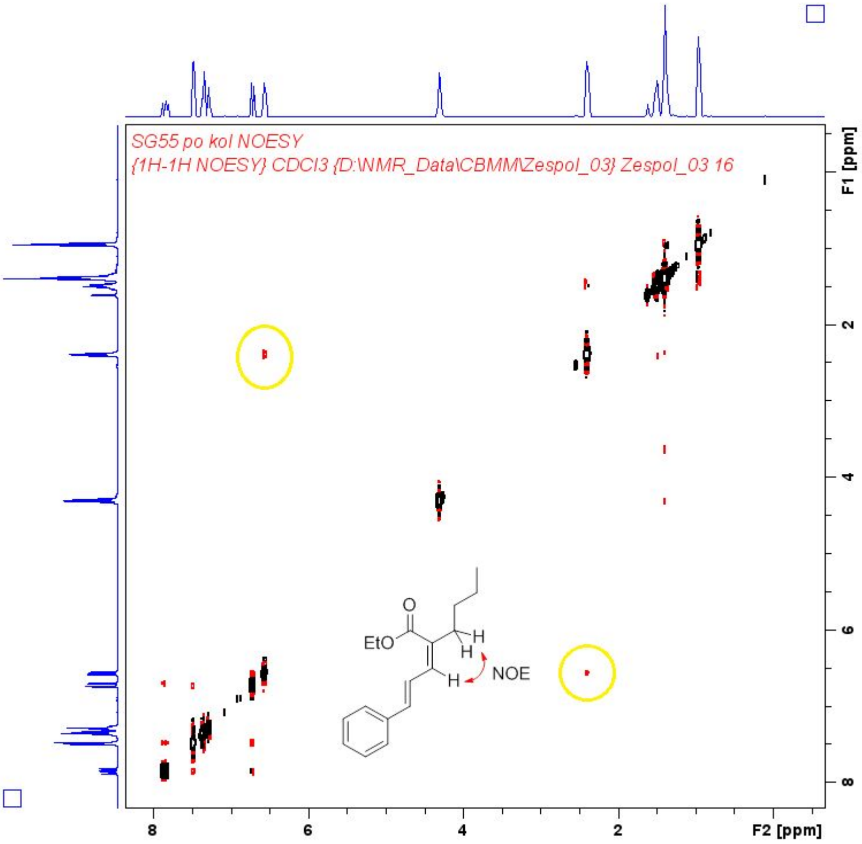

4e

^1H NMR (400 MHz, $CDCl_3$)

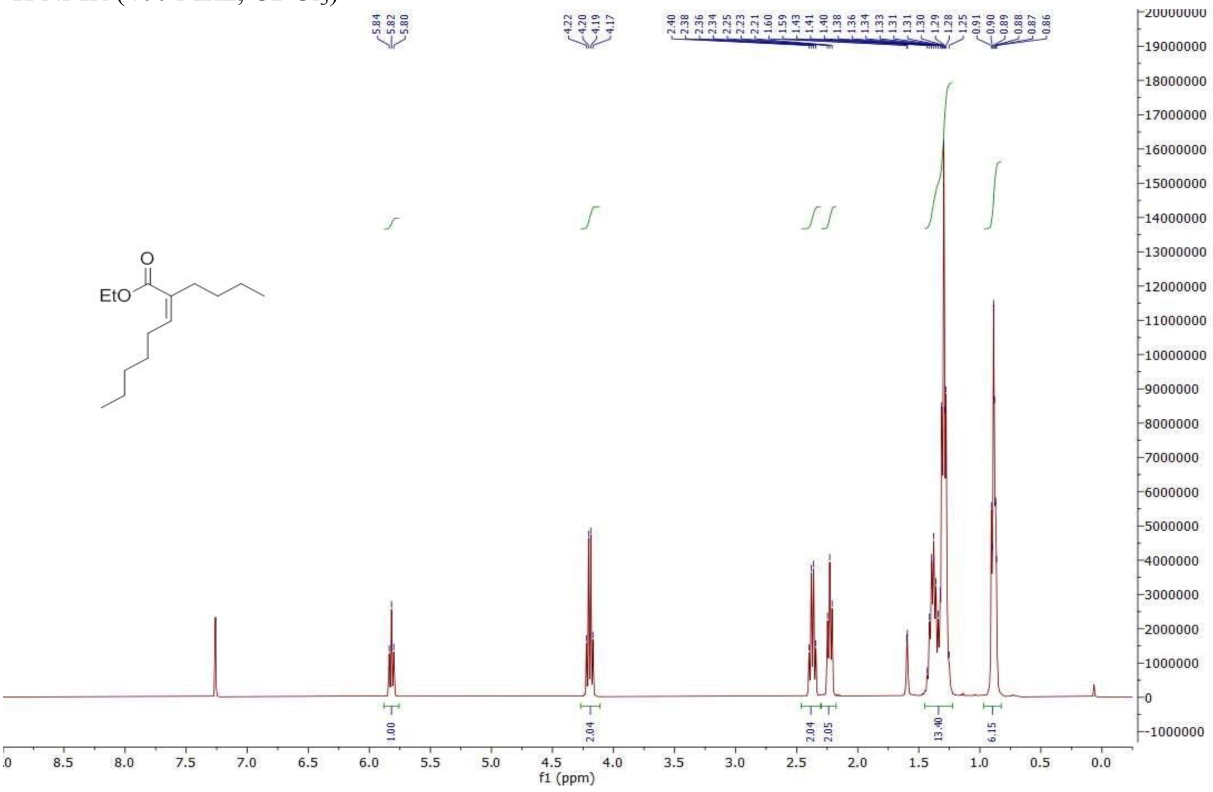

5a

^1H NMR (400 MHz, $CDCl_3$)

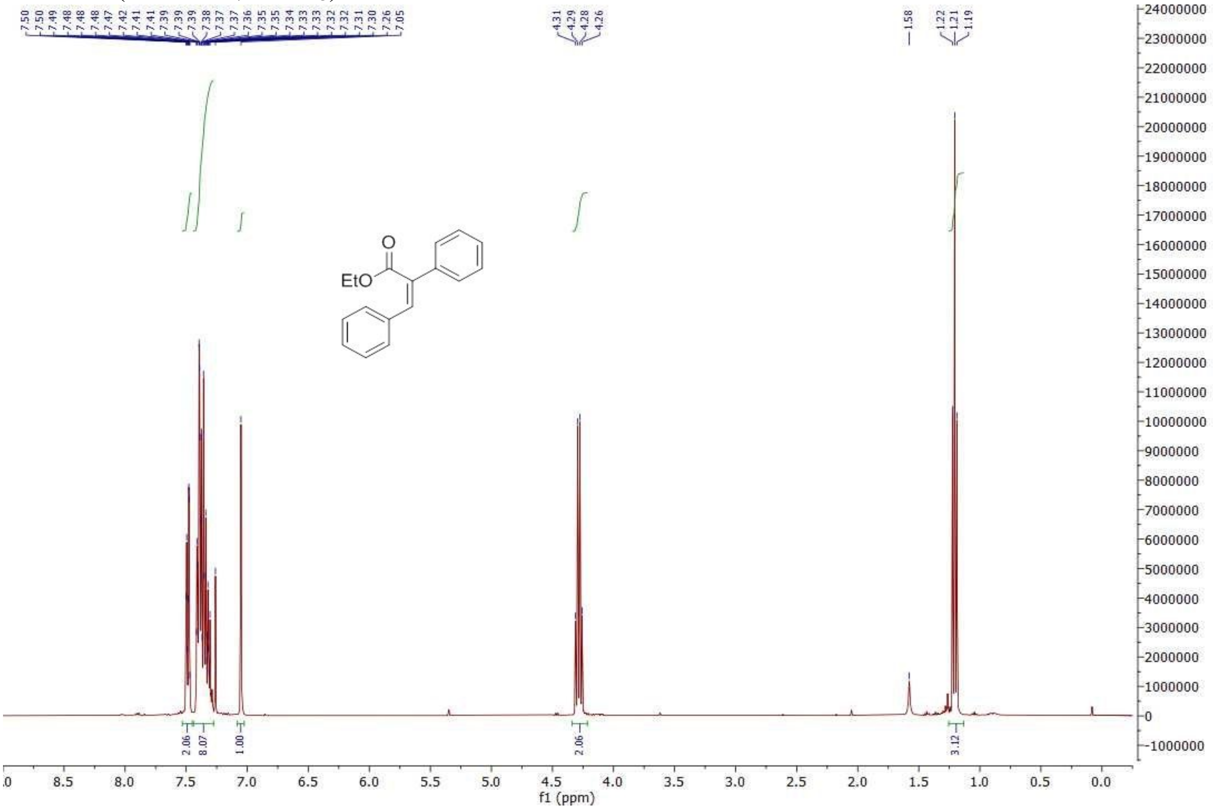

5b

^{1}H NMR (400 MHz, $CDCl_3$)

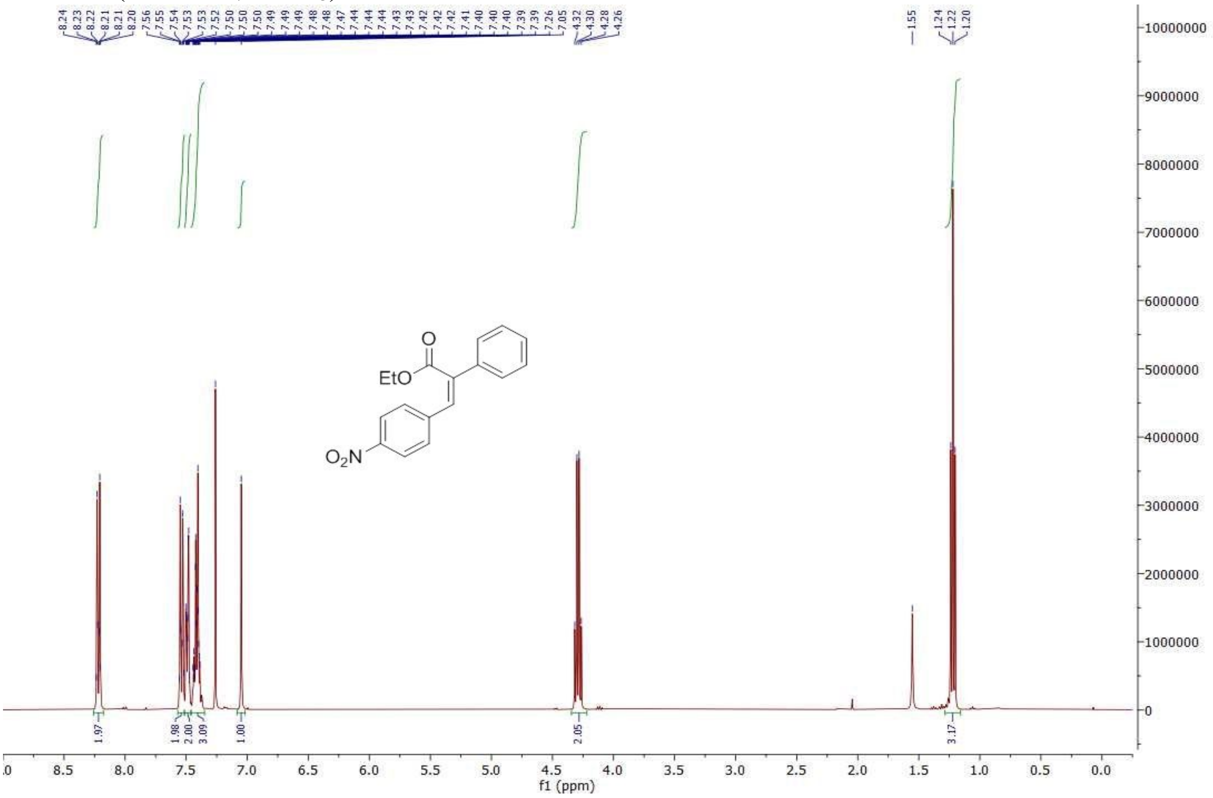

5c

^{1}H NMR (400 MHz, $CDCl_3$)

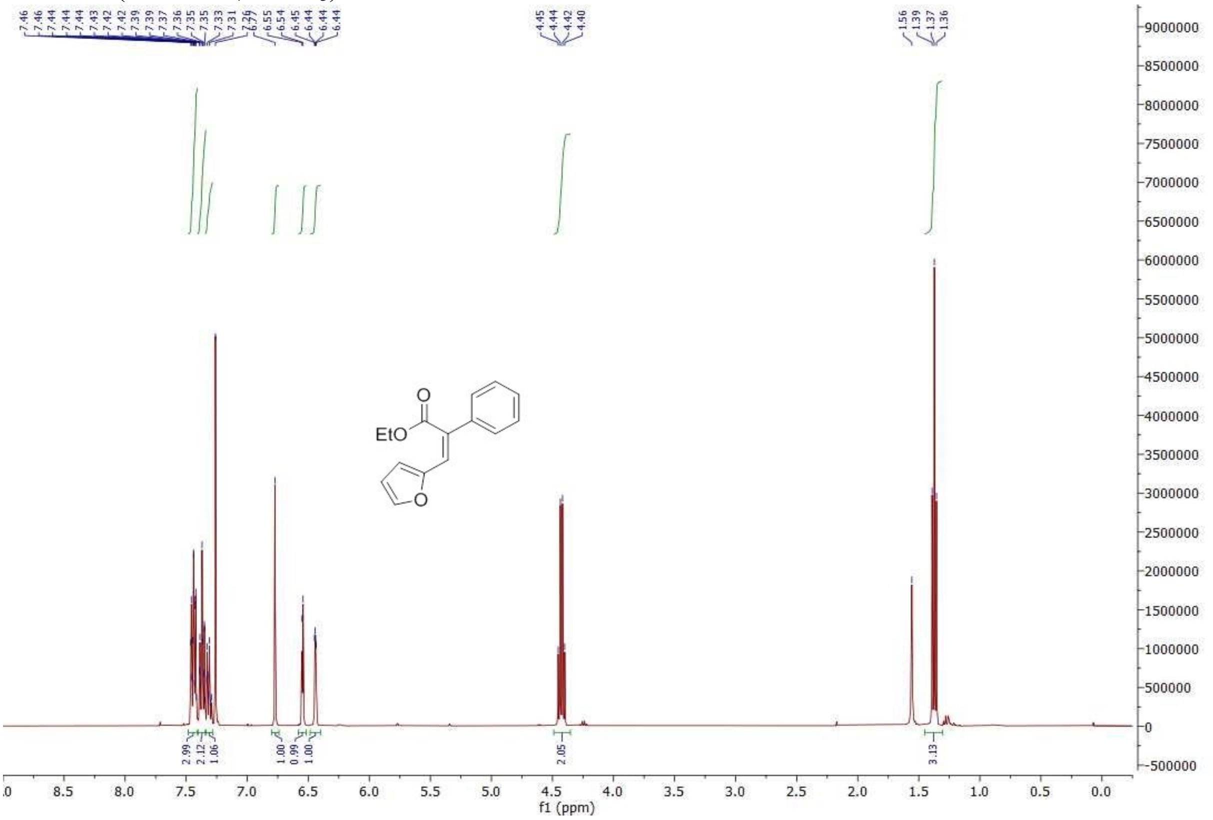

$^{13}C\{^{1}H\}$ NMR (101 MHz, $CDCl_3$)

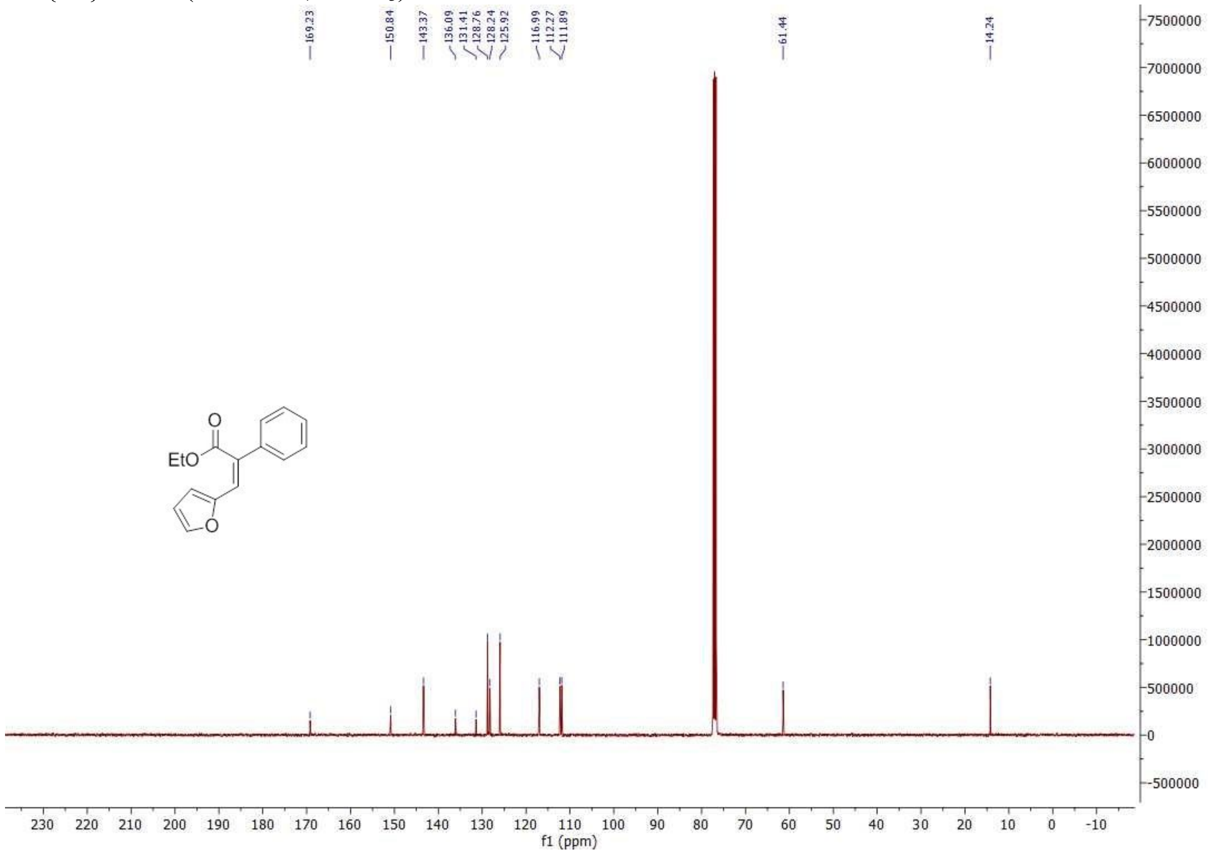

NOESY spectra

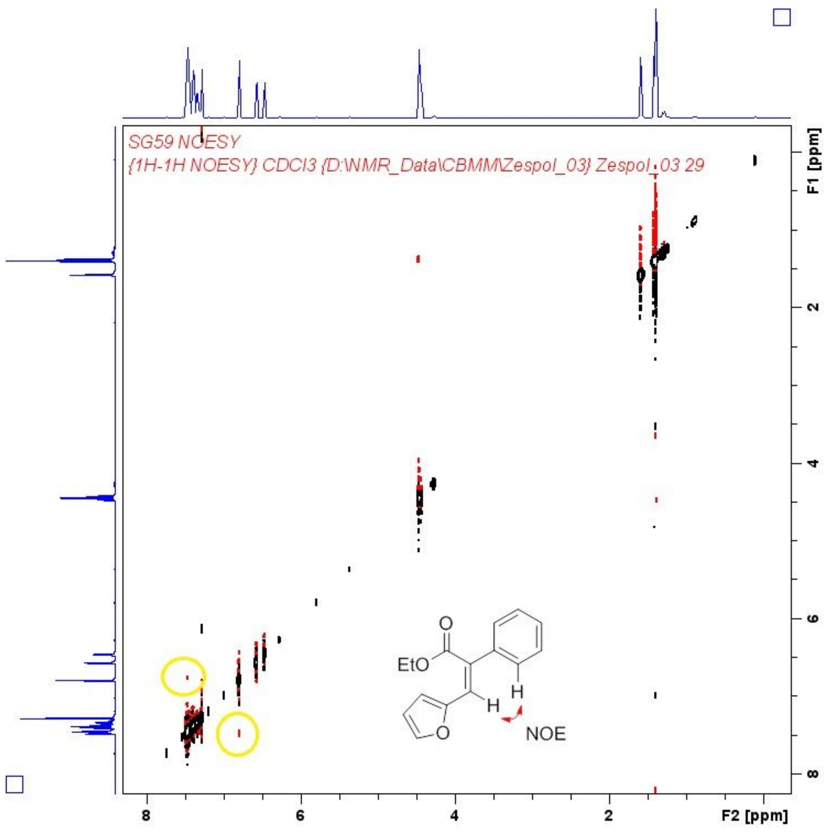

5d

^{1}H NMR (400 MHz, $CDCl_3$)

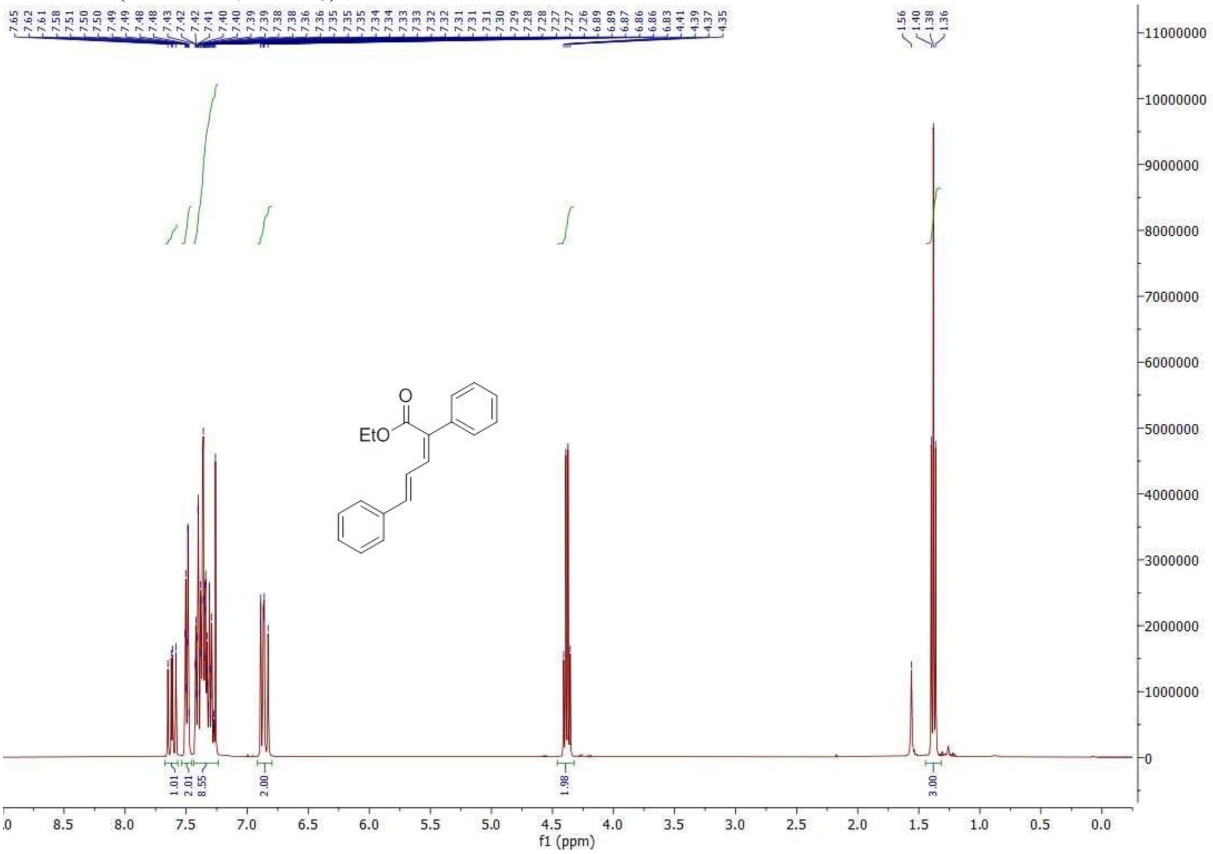

References

1) Janicki, I. Kiełbasiński, P. A straightforward, purification-free procedure for the synthesis of Ando and Still-Gennari type phosphonates. *Synthesis*, **2020**, *54*, 378-382.
2) Murakami, R.; Kojima, N.; Kamo, R.; Tanishima, H.; Inagaki, F. Photoisomerization of alkenes via energy transfer enabled by Cu-acetylide complexes. *Eur. J. Org. Chem.* **2023**, *26*, e202300948
3) Cristau, H. J.; Taillefer, M. Reactivity of substituted and unsubstituted diphenylphosphonium diylides towards carbonic acids derivatives. *Tetrahedron*, **1998**, *54*, 1507-1522.
4) Xavier, T.; Condon, S.; Pichon, C.; Le Gall, E.; Presset, M. Synthesis of α,β-disubstituted acrylates via Galat reaction. *Org. Lett.* **2019**, *21*, 6135-6139.
5) Henin, F.; Mortezaei, R.; Muzart, J.; Pete, J-P.; Piva, O. Photodeconjugaison enantioelective d'esters et de lactones conjuguees en presence d'ephedrine. *Tetrahedron*, **1989**, *45*, 6171-6196.
6) Bargiggia, F.; Piva, O. Anionic versus photochemical diastereoselective deconjugation of diacetone D-glucose α,β-unsaturated esters. *Tetrahedron Asymm.* **2003**, *14*, 1819-1827.
7) Kobayashi, K.; Okamoto, I.; Morita, N.; Kiyotani, T.; Tamura, O. Synthesis of the proposed structure of phaeosphaeride A. *Org. Biomol. Chem.* **2011**, *9*, 5825-5832.
8) Grison, C.; Genève, S.; Halbin, E.; Coutrot, P. Stereoselective synthesis of vinylogous peptides. *Tetrahedron* **2001**, *57*, 4903-4923.
9) Ceccherelli, P; Curini, M.; Marcotullio, M. C.; Rosati, O.; Wenkert, E. Regioselectivity of rhodium(II)-catalyzed decomposition of 1-alkyl-1(diazoacetyl)alkenes. Synthesis of 2-alkyl-2-cyclopentenones and 2-alkylidenecyclopentanones. *J. Org. Chem.* **1991**, *56*, 7065-7070.
10) Zong, Y.; Tang, Y.; Tsui, G. C. Rhodium(I)-catalyzed defluorinative coupling of boronic acids with monofluoroalkenes. *Org. Lett.* **2022**, *24*, 6380-6385.
